# Five-year trajectories of multimorbidity patterns in an elderly Mediterranean population using Hidden Markov Models

**DOI:** 10.1038/s41598-020-73231-9

**Published:** 2020-10-09

**Authors:** Concepción Violán, Sergio Fernández-Bertolín, Marina Guisado-Clavero, Quintí Foguet-Boreu, Jose M. Valderas, Josep Vidal Manzano, Albert Roso-Llorach, Margarita Cabrera-Bean

**Affiliations:** 1 Fundació Institut Universitari per a la recerca a l’Atenció Primària de Salut Jordi Gol i Gurina (IDIAPJGol), Gran Via Corts Catalanes, 587 àtic, 08007 Barcelona, Spain; 2 Universitat Autònoma de Barcelona, Bellaterra (Cerdanyola del Vallès), Spain; 3 Department of Psychiatry, Vic University Hospital, Francesc Pla El Vigatà, 1, 08500 Vic Barcelona, Spain; 4 Health Services & Policy Research Group, Academic Collaboration for Primary Care, University of Exeter Medical School, Exeter, EX1 2LU UK; 5 Signal Theory and Communications Department, Universitat Politècnica de Catalunya, Barcelona Tech., Campus Nord, UPC D5, Jordi Girona 1-2, 08034 Barcelona, Spain

**Keywords:** Diseases, Medical research

## Abstract

This study aimed to analyse the trajectories and mortality of multimorbidity patterns in patients aged 65 to 99 years in Catalonia (Spain). Five year (2012–2016) data of 916,619 participants from a primary care, population-based electronic health record database (Information System for Research in Primary Care, SIDIAP) were included in this retrospective cohort study. Individual longitudinal trajectories were modelled with a Hidden Markov Model across multimorbidity patterns. We computed the mortality hazard using Cox regression models to estimate survival in multimorbidity patterns. Ten multimorbidity patterns were originally identified and two more states (death and drop-outs) were subsequently added. At baseline, the most frequent cluster was the *Non-Specific Pattern* (42%), and the least frequent the *Multisystem Pattern* (1.6%)*.* Most participants stayed in the same cluster over the 5 year follow-up period, from 92.1% in the *Nervous, Musculoskeletal* pattern to 59.2% in the *Cardio-Circulatory and Renal* pattern. The highest mortality rates were observed for patterns that included cardio-circulatory diseases: *Cardio-Circulatory and Renal* (37.1%); *Nervous, Digestive and Circulatory* (31.8%); and *Cardio-Circulatory, Mental, Respiratory and Genitourinary* (28.8%). This study demonstrates the feasibility of characterizing multimorbidity patterns along time. Multimorbidity trajectories were generally stable, although changes in specific multimorbidity patterns were observed. The Hidden Markov Model is useful for modelling transitions across multimorbidity patterns and mortality risk. Our findings suggest that health interventions targeting specific multimorbidity patterns may reduce mortality in patients with multimorbidity.

## Introduction

Multimorbidity, the coexistence of two or more chronic diseases in the same individual, is a growing global concern^1^. The estimated prevalence of multimorbidity in people over 65 years of age reaches 95% in some studies^2^. Population ageing means that multimorbidity rates will dramatically rise in the coming decades^3^. The WHO World Report on Ageing and Health advocates for the characterization of health trajectories to better understand the dynamics of ageing and to optimize well-being and health gains from interventions^4^. This implies measurements of multimorbidity more sophisticated than the usual analyses limited to weighted counts of conditions for estimating the prevalence and incidence of multimorbidity^5^.

The longitudinal characterization of disease patterns is essential for uncovering relationships over time and for the development of prediction models^6^. To date, studies have predominantly focused on diseases rather than persons, since the unit of analysis is the identification of multimorbidity patterns^7–9^^.^. This approach precludes the exploration of the trajectories and evolution of multimorbidity over a persons’ lifetime^7^. By refocusing the analysis on individuals, we can obtain a better characterisation of the population groups with multimorbidity. In turn, we will produce more accurate data for the development of clinical guidelines, and for more targeted preventive, diagnostic, prognostic and treatment strategies^10,11^ .

Most longitudinal analyses have used a cross-sectional design and studies that include the whole longitudinal structure of the data are scarce^12–14^. To define the trajectories of multimorbidity patterns, it is essential to consider the different diseases that affect people over time. Therefore, it is necessary to conduct studies that offer more information on how individual techniques are influenced by specific identification of disease patterns or even test new statistical approaches adapted for the study of multimorbidity.

In recent years, dynamic machine learning methodologies like the Hidden Markov Models (HMM) have been applied to identify multimorbidity patterns. The HMM integrate a dynamic Bayesian network that works with the temporal sequence of the observed patient’s data. In a HMM, the observations are random variables conditioned by a hidden state or cluster. For instance, if it is considered that each patient belongs to one multimorbidity pattern each year; it is not possible to observe the cluster directly. Instead, we have the data contained in the Electronic Health Records (EHR) for each year of study, which are the observed outcomes from the time series. Previous studies have applied dynamic Bayesian networks for health analysis. For instance, a Dutch analysis applied HMM to a large dataset from practices of patients that had events on comorbidities related to atherosclerosis and the discovered hidden clusters were further correlated to medical-oriented outcomes^15^. Other examples relate to the decomposition of shared latent factors using Bayesian multimorbidity dependency maps and to healthcare predictive risk modelling^16,17^. Primary Care EHR, where data are routinely collected, represents a unique resource for the longitudinal analysis of multimorbidity patterns.

The aim of this study is to analyse and describe multimorbidity patterns, their trajectories and mortality over time in people over 65 years of age during the 2012–2016 period in Catalonia using primary care EHR.

## Methods

### Design, setting, and study population

**Figure 1:**
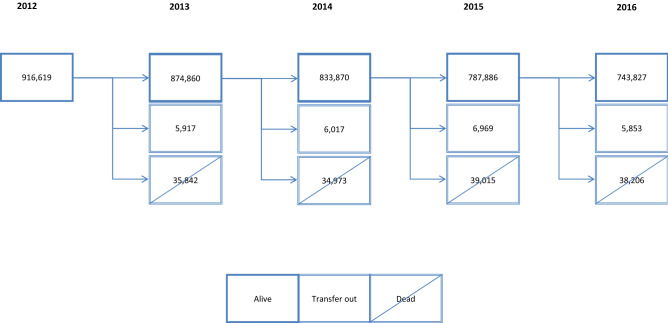
Longitudinal Flow Chart of study period (year 2012–2016; N = 916,619 persons).

A longitudinal study was conducted in Catalonia (Spain), a Mediterranean region of 7,515,398 inhabitants^18^. The Spanish National Health Service provides universal coverage, financed mainly by tax revenue. The Catalan Health Institute (CHI) manages 284 primary health care centres (PHCs) that serve 5,501,784 patients (274 PHCs), which represent 74% of the population; the remaining PHCs are managed by other providers. A total of 916,619 people were included at baseline and 743,827 completed all follow-up (see Fig. 1).

Inclusion criteria consisted of individuals aged 65–99 years on 31 December 2011 that survived until 31 December 2012 (index date), which had consulted their primary care physician at least once during the 5-year study period (2012–2016). No new entries were allowed in the cohort. Attrition was caused by mortality or dropouts due to transfer to other health systems.

### Data sources

The CHI’s Information System for Research in Primary Care (SIDIAP) contains the clinical information as EHR recorded by the PHCs since 2006^19^. The SIDIAP database includes anonymized longitudinal EHR from primary and secondary care that collect information on demographics, symptoms, diagnoses, prescriptions and socio-economic status.

### Variables

All variables were obtained directly from the SIDIAP database.

#### Chronic diseases

Diseases are coded in the SIDIAP using the International Classification of Diseases version 10 (ICD-10). An operational definition of multimorbidity was used based on the selected 60 chronic diseases determined by the Swedish National study of Aging and Care in Kungsholmen (SNAC-K), with additional clinical, lab and drug related parameters for the assessment of certain conditions. Multimorbidity was defined by the presence of two or more diagnoses from the 60 chronic disease categories defined in the SNAC-K study^20^ (see Supplementary 1).

#### Death and drop out

Death was measured as the occurrence of this event, regardless of cause. The start date for calculation of risk of death was 1 January 2013. A person that dropped out of the health system during the study period was considered lost to follow-up (drop-out).

#### Other variables

Additional variables included in the study (baseline and end of study) were socio-demographic variables such as age at baseline (years), sex (men, women), socio-economic status (MEDEA index; quintiles from least to most deprived)^21^, number of invoiced drugs and polypharmacy (five or more different drugs), and number of visits to Primary Care.

### Statistical analysis

Descriptive statistics were used to summarize overall information. Participant’s characteristics and disease prevalence were measured at baseline and final year.

For the grouping of participants, it was assumed that patient population was initially distributed into a fuzzy set of clusters, corresponding to the different multimorbidity patterns^22^. In order to model the temporal evolution of both individuals and clusters, the sequential individual observations were assumed to follow a dynamic random process represented by an HMM so that each cluster was associated with a hidden state or multimorbidity pattern.

For the development of the HMM, all features from all individuals at each point of the follow-up were considered. The HMM was conducted in two stages. In the first stage, the set of features that represent each patient’s EHR for each year was selected. To prevent including statistical noise and spurious findings in the model, diseases with a prevalence < 2% at baseline were excluded (Supplementary 1). The dataset was pre-processed by applying a mixture of Principal Component Analysis (PCA) to the continuous original features and a Multiple Correspondence Analysis (MCA) to the categorical features in order to reduce the number of features on the new dataset. Afterwards, a fuzzy segmentation procedure (Fuzzy C-means algorithm or FCM) was applied on the new dataset to identify an initial set of clusters, which was used to initialize some of the HMM parameters in the next stage. Finally, two more clusters were added with the objective to account for drop-out and death.

In the second stage, the set of HMM parameters, composed of the initial cluster probabilities, the inter cluster transition probabilities and the emission distributions provided by FCM, was fitted into the observation data by applying the Baum-Welch (BW) algorithm. In this process, data originating from each individual formed a short temporal sequence of five available vectors with all their observed features, one vector per year. It was assumed that the different sequences were statistically independent of each other, since each sequence corresponds to a different individual^23^.

Once the model parameters were learned, the longitudinal trajectories followed by the individuals could be inferred. In this process, given each observed sequence, the best cluster trajectory is computed by maximizing the probability of the observed sequence conditioned to the computed model parameters (Viterbi Algorithm). To validate the model, a comparison between BW and Viterbi transition probabilities was conducted, indicating a good agreement between theoretical and observed values.

To optimize the performance of the selected mathematical model, the iterative process involved in the application of the BW algorithm was initialized using different initial values of the parameters to be learned. The best model selected was the model that delivers the highest probability of the available data conditioned to the final estimated model parameters. This procedure is equivalent to applying the Bayes Information Criterion to choose the best obtained set of HMM parameters. Supplementary 2 includes further methodological information on the machine-learning techniques applied to the dataset.

#### Multimorbidity patterns

The final clusters were defined as multimorbidity patterns. To evaluate the consistency and utility of the final clusters, we used clinical criteria from clusters previously described in the literature, together with the consensus opinion of the research team physicians (3 family doctors and 1 epidemiologist). To describe the multimorbidity patterns, percentages of diseases (O) in each cluster were calculated. Observed/expected ratios (O/E-ratios) were obtained by dividing disease prevalence in the cluster by disease prevalence in the overall population. Exclusivity (E), defined as the proportion of patients with the disease included in the cluster over the total number of patients with the disease, was also calculated. A disease was considered to be part of a multimorbidity pattern when the O/E-ratio ≥ 2^24,25^.

In order to account for drop-out and mortality, two more clusters were added. In doing so, data from people who transferred and died ceased to contribute to the dynamic characterization of the patterns after the occurrence of these events.

The Cox proportional-hazards regression models were fitted to estimate the mortality hazard of multimorbidity patterns at baseline. Time to follow-up was the time between index date and all-cause death. People were followed until censored (lost to follow-up or end of observation). Hazard ratios (HR) and 95% confidence intervals (CI) were adjusted for age, sex, and socio-economic status (MEDEA index). Multiple Imputation was used to minimize the selection bias arising from the presence of missing values for MEDEA (7%), while Chained Equations were used to obtain seven imputed datasets. The final models were fitted with the multiple imputation datasets using Rubin’s rules to combine effect estimates and standard errors, thus allowing for the uncertainty related to missing data.

The analyses were carried out using R version 3.5.1 (R Foundation for Statistical Computing, Vienna, Austria). The significance level was set at α = 0.05.

### Ethical considerations

The study followed national and international regulations for research involving human subjects: Declaration of Helsinki Ethical Principles for Medical Research Involving Human Subjects and Good Research Practice principles and guidelines. The protocol of the study was approved by the Clinical Research Ethics Committee, Fundació Institut Universitari per a la recerca a l'Atenció Primària de Salut Jordi Gol i Gurina (IDIAPJGol) (P16/151). All data were anonymized and confidentiality of EHR was guaranteed at all times in accordance with national and international law (Ley Orgánica 3/2018, de 5 de diciembre, de Protección de Datos Personales y garantía de los derechos digitales and General Data Protection Regulation (EU) 2016/679 (GDPR)); thus, it was not necessary to ask for informed consent to the participants.

## Results

In this study, 916,619 individuals were included at baseline (women: 57.7%; mean age: 75.4; standard deviation, SD: 7.4), of which 853,085 (93.1%) met multimorbidity criteria. Follow up was completed by 743,827 participants (Fig. 1). Ten multimorbidity patterns were identified at baseline, and two additional clusters, representing death and dropouts, were added for the five-year analysis.

At baseline, the cluster with the highest proportion of the sample (42%), named *Cluster 1 (C1)-Non-Specific Pattern*, consisted of non-overrepresented diseases such as prostate disease, hypertension, solid neoplasms and dyslipidaemia. The remaining patterns, in descending order of sample size, were named with the most over-represented diseases, for instance, *Cluster 2 (C2)-Eye Impairment and Mental* (19.3%) (See Supplementary 3 for more details).

Women were predominant in the following patterns: *C2-Eye Impairment and Mental* (73.7%), *C4-Cardio-Circulatory and Renal* (70.8%), *C7-Respiratory and Ear* (74.8%), *C9-Nervous, Musculoskeletal and Minor* (92.6%), *C10-Multisystem* (89.9%). The only pattern with clear male predominance was *C5-Cardio-Circulatory, Mental, Respiratory and Genitourinary* (93.3%). The mean age for the oldest pattern was 80.6 years (*C4-Cardio-Circulatory and Renal*), and the youngest pattern had a mean age of 72.8 years (*C9-Nervous, Musculoskeletal and Minor*). In relation to socio-economic status, pattern *C4-Cardio-Circulatory, Mental, Respiratory and Genitourinary* was most prevalent among people living in the most deprived areas (see Supplementary 4 for further details on multimorbidity patterns).

The clusters with a higher median number of chronic diseases were *C10-Multisystem Pattern* (11 diseases); *C4-Cardio-Circulatory and Renal* and *C9-Nervous, Musculoskeletal and Minor* (9 diseases); and *C5-Cardio-Circulatory, Mental, Respiratory and Genitourinary* (8 diseases). Considering all clusters, the median of diseases ranged from 4 to 11. Three clusters presented the highest number of polymedicated patients, with a median of 8 prescribed drugs: *C4-Cardio-Circulatory and Renal* (86.0%); *C10-Multisystem Pattern* (81.4%); and *C5-Cardio-Circulatory, Mental, Respiratory and Genitourinar*y (80.7%). The median of visits ranged from 7 to 20. The three clusters with more median visits were: (1) *C4-Cardio-Circulatory and Renal* (20 visits); (2) *C10-Multisystem Pattern* (15 visits); (3) *C5-Cardio-Circulatory, Mental, Respiratory and Genitourinary* (14 visits). Toward the end of the study, *C4-Cardio-Circulatory and Renal* and *C5 Cardio-Circulatory, Mental, Respiratory and Genitourinary* increased the median number of visits (22.0 and 15.0, respectively). The remaining clusters maintained the same number of visits at the beginning and at the end of the study period (Supplementary File 4). On the other hand, ten clusters showed an equal median number of drugs (ranging from 3 to 8) at the beginning and end of the study (Supplementary File 4). The clusters presenting higher mortality were: *C4-Cardio-Circulatory and Renal* (37.1*%); C6-Nervous, Digestive and Circulatory* (31.8%); and *C5-Cardio-Circulatory, Mental, Respiratory and Genitourinary* (28.8%). The proportion of deaths ranged from 3.3% (*C9-Nervous, Musculoskeletal and Minor*) to 37.1% (*C4-Cardio-Circulatory and Renal*) (Table 1).

**Table 1:** Transition probabilities for Viterbi decoding from start to end of study.

Origin\Dest	C1—Non-Specific	C2—Eye Impairment and Mental	C3—Minority Metabolic Autoimmune-Inflammatory	C4—Cardio-Circulatory and Renal	C5—Cardio-Circulatory, Mental, Respiratory and Genitourinary	C6—Nervous, Digestive and Circulatory	C7—Respiratory and Ear	C8—Digestive	C9—Nervous, Musculoskeletal and Minor	C10—Multisystem Pattern	Dropout	Death
C1—Non-Specific	**66.1%**	**3.5%**	**4.2%**	1.7%	2.0%	**2.6%**	1.1%	1.2%	0.5%	0.1%	**3.2%**	**13.7%**
C2—Eye Impairment and Mental	0.3%	**79.0%**	0.7%	2.0%	0.7%	1.9%	1.0%	0.9%	**2.1%**	1.5%	**2.0%**	**7.9%**
C3—Minority Metabolic Autoimmune-Inflammatory	1.3%	0.1%	**76.7%**	0.6%	0.3%	1.1%	0.5%	0.7%	0.1%	0.3%	**2.1%**	**16.1%**
C4—Cardio-Circulatory and Renal	0.1%	0.0%	0.0%	**59.2%**	0.0%	0.3%	0.1%	0.2%	0.0%	0.1%	**2.9%**	**37.1%**
C5—Cardio-Circulatory, Mental, Respiratory and Genitourinary	0.2%	0.0%	0.1%	0.0%	**67.3%**	0.8%	0.0%	0.5%	0.0%	0.0%	**2.3%**	**28.8%**
C6—Nervous, Digestive and Circulatory	1.5%	0.5%	0.2%	0.1%	0.2%	**61.2%**	0.2%	0.1%	0.1%	0.1%	**4.0%**	**31.8%**
C7—Respiratory and Ear	1.7%	1.4%	0.4%	0.5%	0.3%	0.7%	**77.9%**	0.2%	0.4%	0.9%	**2.1%**	**13.7%**
C8—Digestive	**2.2%**	1.4%	0.5%	0.3%	0.3%	0.5%	0.2%	**68.4%**	0.1%	0.4%	**2.5%**	**23.3%**
C9—Nervous, Musculoskeletal and Minor	0.3%	0.7%	0.1%	0.4%	0.0%	0.5%	0.3%	0.1%	**92.1%**	1.0%	1.1%	**3.3%**
C10—Multisystem Pattern	0.1%	0.4%	0.0%	0.1%	0.0%	0.1%	0.2%	0.0%	0.0%	**85.4%**	1.9%	**11.6%**
Dropout	0.0%	0.0%	0.0%	0.0%	0.0%	0.0%	0.0%	0.0%	0.0%	0.0%	**100.0%**	0.0%
Death	0.0%	0.0%	0.0%	0.0%	0.0%	0.0%	0.0%	0.0%	0.0%	0.0%	0.0%	**100.0%**

Bold show probabilities higher than 2%.

**Figure 2:**
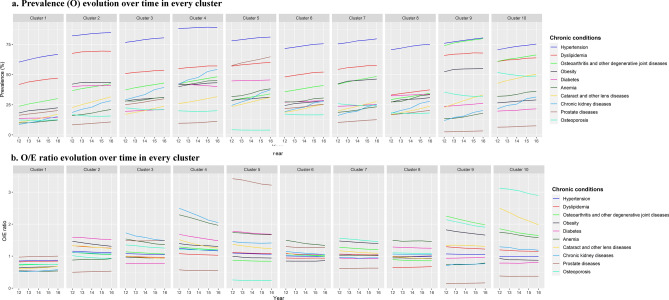
Prevalence and O/E ratio evolution over time in every cluster. (**a**) Prevalence (O) evolution over time in every cluster. (**b**) O/E ratio evolution over time in every cluster.

The evolution during follow up of O/E ratio and prevalence (O) for the ten most prevalent chronic diseases shown in Fig. 2 indicates that O/E ratio maintains stable or slightly decreases over time and prevalence increases over time for all multimorbidity patterns.

Table 1 shows transition probability between clusters. The transition matrix diagonal shows that a high proportion of patients remain in the same cluster over the follow-up period. Permanence in the same pattern is highest (92.1%) in the *C9-Nervous, Musculoskeletal and Minor* pattern, and lowest (59.2%) for patients starting in the *C4-Cardio-Circulatory and Renal* pattern. The patterns with highest mortalities were *C5-Cardio-Circulatory, Mental, Respiratory and Genitourinary*; *C6-Nervous, Digestive and Circulatory*; and *C7-Respiratory and Ear*.

**Figure 3:**
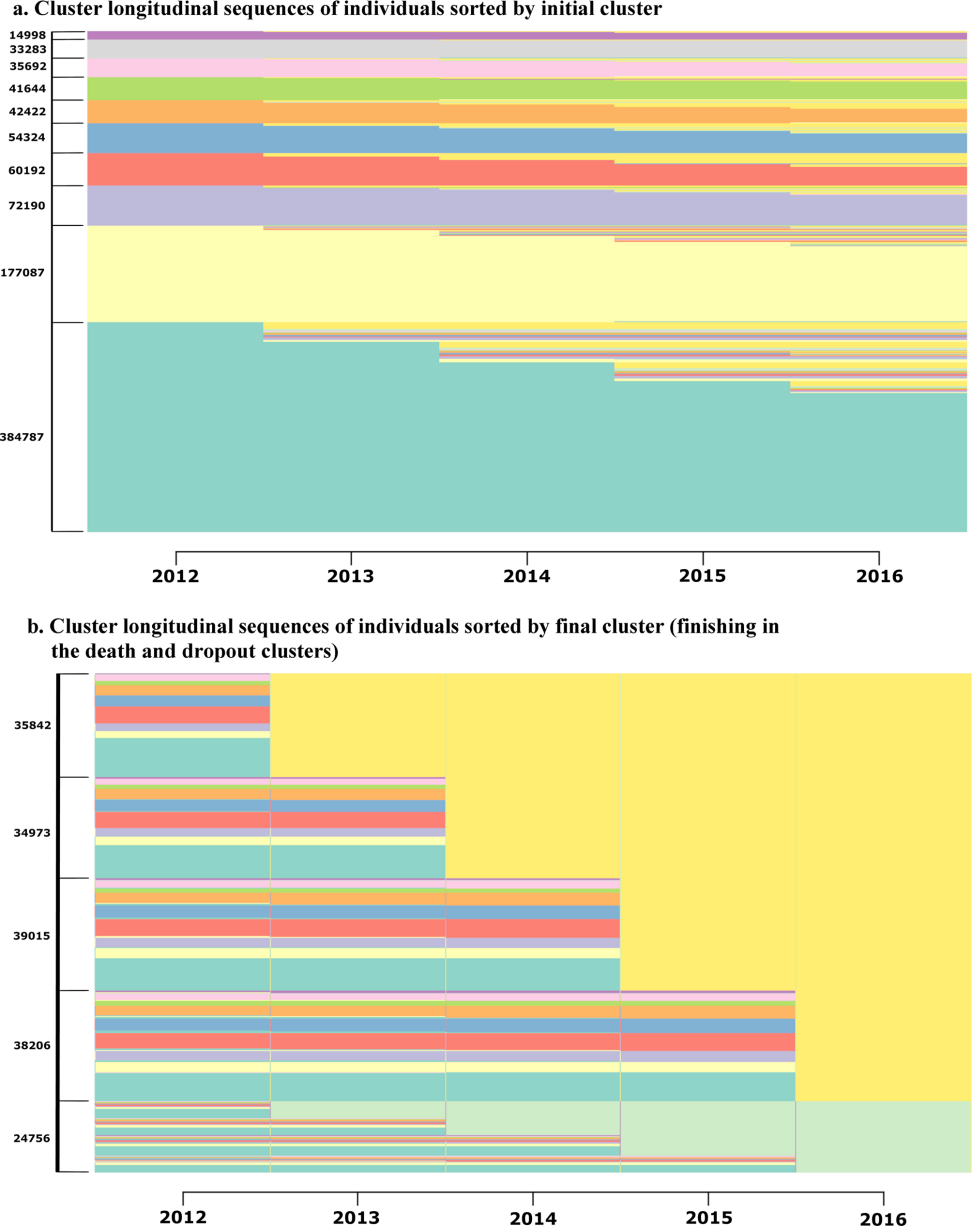
Cluster longitudinal sequences of individuals. (**a**) Cluster longitudinal sequences of individuals sorted by initial cluster. (**b**) Cluster longitudinal sequences of individuals sorted by final cluster (finishing in the death and dropout clusters).

Transitions and trajectories are shown in Fig. 3. Figure 3a shows the individual cluster longitudinal sequences sorted by initial cluster and the number of patients corresponding to each initial cluster, while Fig. 3b shows the cluster longitudinal sequences of individuals that finished in the drop-out or death clusters sorted by final cluster. Most trajectories consisted in a single transition between two clusters. The most frequent transitions were to the death and drop out clusters (Fig. 3b). The most likely transitions to other clusters corresponded from *C1-Non-Specific* to *C3-Minority Metabolic Autoimmune-Inflammatory* (4.2%) and from *C1* to *C2-Eye Impairment and Mental* (3.5%) (Table 1).

**Figure 4:**
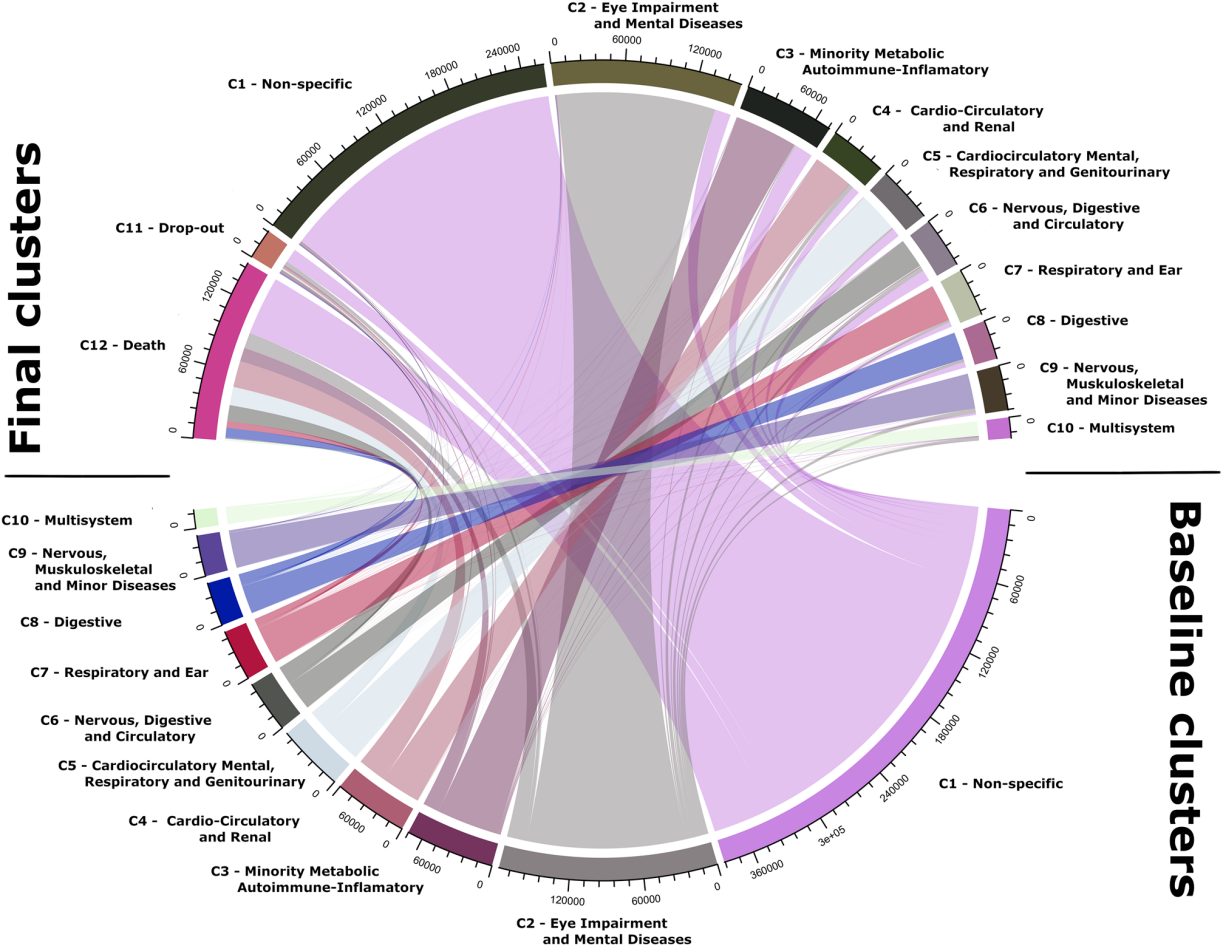
Transition from Baseline (B) to Final (F).

Figure 4 also shows the transition between baseline and final clusters. The proportion of sample remains stable for each baseline pattern, except for the *C1-Non-Specific* pattern, which shows a decrease from 42 to 28%.

The study of the mortality showed that *C4-Cardio-Circulatory and Renal; C5 Cardio-Circulatory, Mental, Respiratory and Genitourinary; C6-Nervous, Digestive and Circulatory;* and *C8-Digestive* presented the highest hazard of death after adjusting for confounders (adjusted HRs over 1.5) (Table 2).

**Table 2:** Cox proportional-hazards regression for mortality cluster.

C1—Non-Specific	Death								
	Unadjusted			Adjusted^a^			Adjusted^b^		
	HR	2.5%	97.5%	HR	2.5%	97.5%	HR	2.5%	97.5%
C2—Eye Impairment and Mental	0.549	0.539	0.559	0.608	0.595	0.622	0.645	0.632	0.657
C3—Minority Metabolic Autoimmune-Inflammatory	1.227	1.202	1.251	1.015	0.992	1.038	1.064	1.043	1.086
C4—Cardio-Circulatory and Renal	3.373	3.322	3.426	1.978	1.942	2.014	2.086	2.053	2.119
C5—Cardio-Circulatory, Mental, Respiratory and Genitourinary	2.404	2.362	2.447	1.656	1.622	1.692	1.757	1.725	1.790
C6—Nervous, Digestive and Circulatory	2.792	2.740	2.845	1.937	1.896	1.979	1.982	1.945	2.020
C7—Respiratory and Ear	1.038	1.011	1.067	1.031	0.999	1.064	1.102	1.072	1.132
C8—Digestive	1.836	1.794	1.879	1.841	1.791	1.891	1.969	1.924	2.016
C9—Nervous, Musculoskeletal and Minor	0.229	0.216	0.243	0.301	0.280	0.324	0.356	0.335	0.377
C10—Multisystem Pattern	0.890	0.850	0.932	0.684	0.646	0.724	0.812	0.775	0.851

*HR* hazard ratio.

^a^Sex, Age, MEDEA (Complete Cases).

^b^Sex, Age, MEDEA (Multiple Imputation).

## Discussion

### Key results

Most studies on the trajectories of multimorbidity have included small samples and few diseases and have not used methods able to identify change from multimorbidity patterns over a period of time^26–28^. In this study conducted with data from primary care EHR, we provide longitudinal information on the multimorbidity trajectory of individual patients from a large, representative dataset. The results obtained inform about the direction and magnitude of multimorbidity patterns, on the evolution of diseases in each pattern and on temporal trends. Initially, we obtained the following ten multimorbidity patterns: *Non-Specific; Eye Impairment and Mental Minority Metabolic Autoimmune-Inflammatory; Cardio-Circulatory and Renal; Cardio-Circulatory, Mental, Respiratory and Genitourinary; Nervous, Digestive and Circulatory; Respiratory and Ear; Digestive; Nervous, Musculoskeletal and minor;* and finally, the *Multisystem Pattern.*

A three-stage process was conducted: firstly, we identified the diseases more frequently associated to a multimorbidity pattern using O/E ratio and exclusivity; secondly, we determined the multimorbidity trajectory of a patient; thirdly, we observed that most people remained in the same multimorbidity pattern over time and that multimorbidity increases with age and with number of diseases, specifically hypertension, dyslipidaemia, obesity and diabetes, included in most multimorbidity patterns.

### Interpretation of multimorbidity patterns and trajectories

The use of the O/E ratio and exclusivity presents a complementary approach to understanding the role of each disease in each multimorbidity pattern. The O/E ratio provides information on the most overrepresented disease in each cluster. Exclusivity indicates the proportion of patients with the disease in a specific cluster over the total number of patients with the disease. The overrepresented diseases are the nuclear diseases in each pattern and represent the diseases that characterize people in this cluster. With the use of O/E ratios, the analysis is less influenced by diseases such as hypertension and dyslipidaemia, which are highly prevalent in the whole population. Regarding the 10 most prevalent chronic diseases, their O/E ratio was stable or slightly decreased over time as shown in Fig. 2. The overrepresented diseases are the nuclear diseases in each pattern^22^ and represent the diseases that characterize people in this cluster regardless of the most prevalent diseases. If the exclusivity value is considered, the percentage of people with a certain disease in a specific cluster can be identified. Consequently, the use of O/E ratio and exclusivity indicate which conditions are most likely to co-exist. Therefore, if we look at diabetes as a prevalent disease, we can observe in the *C2-Eye Impairment and Mental* diseases pattern that some diseases such as Glaucoma, Other Eye Diseases and Neurotic, Stress-Related and Somatoform Diseases are nuclear diseases. These diseases correspond to 39.92% of people in this pattern, which is the most frequent pattern for diabetic persons (exclusivity 30.67%). Moreover, this aspect is maintained over the study period, demonstrating that this method describes both the evolution of the pattern characteristics over time and the potential risk/progression of certain diseases^29,30^. Then, we could identify diabetic people who belongs to a specific pattern, C2, and observed the evolution over time, for example.

Up to 41.97% of the study population is included in the *C1-Non-Specific* cluster, whereas only 1.63% of the study population is ascribed to the *C10-Multisystem* pattern. In reality, these two patterns are two sides of the same coin: C1 for the elderly with no severe complications of their multimorbidity (the majority of diseases are cardiovascular risk factors), and C10 for the elderly who are already suffering from serious complications. Importantly, the *C1-Non-Specific* cluster is the pattern with the youngest and least multimorbid patients. Other studies agree that the younger elderly are less frequently diagnosed with multiple diseases^31^. Interestingly, this same *C1-Non-Specific* cluster presented the highest percentage of transition, particularly to *C3-Minority Metabolic Autoimmune-Inflammatory* and *C2-Eye Impairment and Mental*. The transition to the *C2-Eye Impairment and Mental* and *C3-Minority Metabolic Autoimmune-Inflammatory* cluster is supported by the increasing incidence of autoimmune diseases with ageing^32–34^.

The patterns that resulted in a higher burden of care, i.e., those with the greater number of polymedicated people (median prescription of 8 drugs) and the major number of visits (14 to 20 visits per year), were *C4-Cardio-Circulatory and Renal*, *C10-Multisystem Pattern*, and *C5-Cardio-Circulatory, Mental, Respiratory and Genitourinary*. Importantly, *C4- Cardio-Circulatory and Renal* and *C5- Cardio-Circulatory, Mental, Respiratory and Genitourinary* are among the three patterns with the highest mortality. The financial expense and high care burden of these three patterns pose a challenge to health services.

The transition of the persons included in Cluster 1 to *C2-Eye Impairment and Mental Diseases* can be explained through hypertension, which can present long term complications such as hypertension retinopathy and glaucoma. The transition from C1 to the *C3-Minority Metabolic Autoimmune-Inflammatory cluster* should be interpreted under the immunosenescence process, a remodelling of the innate and adaptive system associated with ageing^31–33,35^.

No relevant transitions, in terms of probability values, are observed in patients from other clusters. The probability of remaining in the same pattern during a 5-year period is over 59% in people over 65 years of age. This means that the initial information will determine preventive and management planning for these patients.

### Interpretation of mortality

Patterns *C4-Cardio-Circulatory and Renal; C5-Cardio-Circulatory, Mental, Respiratory and Genitourinary; C6-Nervous, Digestive and Circulator;* and *C7-Respiratory and Ear,* which included cardiovascular, neuropsychiatric, digestive and respiratory diseases were independently associated with a higher mortality risk. These findings are consistent with vital statistics reported for Spain, where digestive and respiratory are the third and cardiovascular diseases are the first cause of death, respectively^36^. These results also agree with studies of large databases that followed patients over a 20-year period^37^.The highest mortality found in pattern *C5-Cardio-Circulatory, Mental, Respiratory and Genitourinary* might be attributable to a higher prevalence of cardiovascular disease in patients with mental health disorders and dementia^36^, sometimes caused by adverse effects of their pharmacological treatment. It has also been hypothesised that the prevalence of mental disorders is higher in patients with cardio-circulatory diseases^38^.

### Comparison with the literature

Comparison with other studies is difficult, since most studies on multimorbidity patterns use cross-sectional instead of longitudinal data^1,39^. Analogy of the results of multimorbidity patterns with other studies can be intricate by the methods, data sources and structures, populations and diseases analyzed. Nevertheless, similarities with other authors can be found. The non-specified pattern is the one most replicated in the literature, for example, Prados-Torres et al. who employed an exploratory factor analysis^40^ and our group with k-means^12^ and fuzzy c-means cluster analysis^41^. In these last articles it can be verified that there is a non-negligible coincidence between the multimorbidity patterns. For example with k-means, we identified six multimorbidity patterns: Musculoskeletal, Endocrine-Metabolic, Digestive/Digestive-Respiratory, Neuropsychiatric, Cardiovascular, and Non-Specific patterns^12^.

In the absence of a universal consensus list for the definition of multimorbidity, we used the list created by the SNAC-K study, which establishes 60 chronic diseases as an operational measure of multimorbidity. The identification of these key relevant diseases has been elaborated by an international and multidisciplinary team (geriatricians, general practitioners and epidemiologists), with the purpose of monitoring multimorbidity and to allow comparison of multimorbidity among countries^20^. In our study, the high number of diseases included in the analysis has resulted in a higher percentage of multimorbidity when compared with other observational studies which only included 4 to 40 diseases^39^.

### Strengths and limitations

This study is based on a large, high-quality database containing primary care records representative of the population with multimorbidity in Catalonia^19^. In addition, we used a validated, clinically driven methodology to measure chronic diseases, which allows a standardized evaluation of chronic diseases in the European Union^20^. On the other hand, the EHR used often have incomplete data, since they are mainly designed to support clinical practice^42^.

Different initializations can be considered in the HMM and there is no guarantee of reaching a global optimum solution, since HMM obtains a local optimum instead. To minimize this effect and to eventually use the model with the greatest likelihood, we performed 100 Baum-Welch realizations with different initializations. In addition, the HMM is most effective for long periods of time. To address this limitation and increase the reliability of the model, the data from all patients was used for every time interval^15^. Furthermore, the model’s suitability to the dataset has been validated by clinical criteria and analytical verification of the likelihood of the model, as explained in the Supplementary 2.

### Implications for practice, policy and research

The longitudinal multimorbidity patterns obtained with HMM methods provide a comprehensive approach to the evolution of multimorbidity over a patient’s lifetime. Our data can predict the pattern in which an elderly person will be in the next 5 years. Importantly, many diseases identified have shared risk factors and consequently preventative interventions in these chronic diseases could alter many trajectories and even shift causes of mortality.

We should underline that 42% of the population ≥ 65 years were included in the *C1-Non-Specific* pattern, which in reality encompasses the most prevalent diseases in the elderly. Moreover, since patients in this pattern typically transition to other multimorbidity patterns associated with higher mortality, they should be specifically targeted for preventive interventions.

We can characterize individuals according to the diseases overrepresented in a specific multimorbidity pattern and assume that they have common etiological, genetic and social determinants. The challenge now is to divert healthcare policy from individual diseases to a personalized multimorbidity pattern.

Characterization of multimorbidity patterns using HMM can be expanded, for instance aggregating information from other health determinants such as fragility. Adding new factors will allow the creation of risk algorithms that can be integrated into the EHR to predict future diseases for each individual, opening the door to patient-tailored prevention and management.

Further studies need to elucidate the mechanisms underlying the various multimorbidity patterns. This is one of the first articles analysing the longitudinal evolution of multimorbidity patterns. These same methods should be applied to younger populations for a longer follow-up period to better characterise the patterns and determinants of multimorbidity.

## Conclusions

Four main aspects differentiate and characterise the ten multimorbidity patterns observed: (1) the proportion of women was higher in C2-*Eye Impairment and Mental; C4-Cardio-circulatory and Renal*; C7-*Respiratory and Ear;* and *C9-Nervous, Musculoskeletal, and Minor,* whereas men predominated in *C5-Cardio-circulatory, Mental, Respiratory and Genitourinary*; (2) the highest mortality rates were observed for C4-*Cardio-Circulatory and Renal*; C6-*Nervous, Digestive and Circulatory* and C5-*Cardio-Circulatory, Mental, Respiratory and Genitourinary*; (3) trajectories were generally stable, although changes were common in certain patterns of multimorbidity; (4) most trajectories consisted of a single transition during 5 years of follow up.

This study sheds light on the changing prevalence of multimorbidity patterns over time and on mortality associated with specific patterns. These data are essential for the provision of adequate healthcare for patients with multiple chronic conditions. Future studies on the trajectory of multimorbidity patterns should follow up a younger population for a longer period of time.

## Supplementary information

Supplementary Information.

## Data Availability

The datasets are not available, as researchers have signed an agreement with the Information System for the Development of Research in Primary Care (SIDIAP) concerning confidentiality and security of the dataset, which forbids providing data to third parties. This organization is subject to periodic audits.
