# Supplementary material for: Five-year trajectories of multimorbidity patterns in an elderly Mediterranean population using Hidden Markov Models
Source: Sci Rep. 2020 Oct 9;10:16879. doi: 10.1038/s41598-020-73231-9 (PMC7547668; doi:10.1038/s41598-020-73231-9)
Supplement: Supplementary file 1 — Supplementary Information. [file 41598_2020_73231_MOESM1_ESM.pdf]

Five-year trajectories of multimorbidity patterns in an elderly Mediterranean population using Hidden Markov Models

Concepción Violán[1,2]*, Sergio Fernández-Bertolín[1,2],Marina Guisado-Clavero[1,2], Quintí Foguet-Boreu[1,2,3], Jose M Valderas[4], Josep Vidal Manzano[5], Albert Roso-Llorach[1,2]**, Margarita Cabrera-Bean [5]**.

1. Fundació Institut Universitari per a la recerca a l'Atenció Primària de Salut Jordi Gol i Gurina (IDIAPJGol), Gran Via Corts Catalanes, 587 àtic, 08007 Barcelona, Spain.

2. Universitat Autònoma de Barcelona, Bellaterra (Cerdanyola del Vallès), Spain.

3. Department of Psychiatry, Vic University Hospital. Francesc Pla el Vigatà, 1, 08500 Vic, Barcelona, Spain.

4. Health Services & Policy Research Group, Academic Collaboration for Primary Care, University of Exeter Medical School, Exeter, EX1 2LU, United Kingdom.

5. Signal Theory and Communications Department, Universitat Politècnica de Catalunya, Barcelona Tech. Campus Nord, UPC D5, Jordi Girona 1-2, 08034-Barcelona, Spain.

** Last shared authorship

Corresponding author:

Concepción Violán. Fundació Institut Universitari per a la recerca a l'Atenció Primària de Salut Jordi Gol i Gurina (IDIAPJGol),

Gran Via Corts Catalanes, 587 àtic, 08007 Barcelona, Spain.

Telephone: 0034 93 482 41 24. Fax: 0034 93 482 41 74.

Web page: www.idiapjgol.org E-mail: cviolan@idiapjgol.org

Supplementary 1

Prevalence of the 60 chronic diseases included in the study in individuals aged 65-99 years (N= 916 619, Catalonia, 2012). In three last columns, list of diseases included by prevalence cut off (1%, 2%, All)

Rank	*Chronic conditions*	*Frequency*	*Percentage (%)*	*All diseases included*	*1%*	*2%*
1	Hypertension	650 899	71.0			
2	Dyslipidaemia	466 585	50.9			
3	Osteoarthritis and other degenerative joint diseases	300 803	32.8			
4	Obesity	262 888	28.7			
5	Diabetes	230 460	25.1			
6	Anaemia	167 577	18.3			
7	Cataract and other lens diseases	156 622	17.1			
8	Chronic kidney diseases	153 756	16.8			
9	Prostate diseases	153 635	16.8			
10	Osteoporosis	151 847	16.6			
11	Depression and mood diseases	148 751	16.2			
12	Solid neoplasms	137 045	15.0			
13	Colitis and related diseases	131 512	14.4			
14	Venous and lymphatic diseases	126 997	13.9			
15	Other musculoskeletal and joint diseases	124 765	13.6			
16	Dorsopathies	124 603	13.6			
17	Neurotic, stress-related and somatoform diseases	123 395	13.5			
18	COPD, emphysema, chronic bronchitis	109 603	12.0			
19	Ischemic heart disease	95 434	10.4			
20	Deafness, hearing impairment	90 261	9.9			
21	Sleep disorders	88 739	9.7			
22	Thyroid diseases	88 445	9.7			
23	Other genitourinary diseases	85 468	9.3			
24	Cerebrovascular disease	80 264	8.8			
25	Atrial fibrillation	80 247	8.8			
26	Esophagus, stomach and duodenum diseases	80 043	8.7			
27	Heart failure	74 077	8.1			
28	Other eye diseases	68 939	7.5			
29	Glaucoma	66 162	7.2			
30	Inflammatory arthropathies	62 450	6.8			
31	Dementia	59 213	6.5			
32	Cardiac valve diseases	52 100	5.7			
33	Peripheral neuropathy	49 127	5.4			
34	Other psychiatric and behavioural diseases	46 841	5.1			
35	Asthma	43 663	4.8			
36	Allergy	40 394	4.4			
37	Autoimmune diseases	39 350	4.3			
38	Ear, nose, throat diseases	38 752	4.2			
39	Peripheral vascular disease	30 674	3.4			
40	Other neurological diseases	28 541	3.1			
41	Chronic pancreas, biliary tract and gallbladder diseases	27 321	3.0			
42	Migraine and facial pain syndromes	25 999	2.8			
43	Bradycardias and conduction diseases	25 476	2.8			
44	Chronic liver diseases	22 633	2.5			
45	Other digestive diseases	22 022	2.4			
46	Parkinson and parkinsonism	20 833	2.3			
47	Other metabolic diseases	18 997	2.1			
48	Other cardiovascular diseases	16 833	1.8			
49	Other skin diseases	15 363	1.7			
50	Chronic ulcer of the skin	13 869	1.5			
51	Blood and blood forming organ diseases	13 575	1.5			
52	Other respiratory diseases	9974	1.1			
53	Epilepsy	8981	1.0			
54	Haematological neoplasms	8174	0.9			
55	Chronic infectious diseases	6647	0.7			
56	Inflammatory bowel diseases	5549	0.6			
57	Schizophrenia and delusional diseases	4792	0.5			
58	Blindness, visual impairment	4772	0.5			

59	Multiple sclerosis	576	0.1		[redacted]
60	Chromosomal abnormalities	77	0.0		

Abbreviations: COPD: Chronic obstructive Pulmonary Disease.

the dataset

This annex is devoted to describing the procedure followed to obtain the set of multimorbidity patterns that characterize the patient population and to identify the longitudinal trajectories along the set of most frequent patterns.

Initial dataset selection procedure

The initial database was composed of the registered diagnosis of 60 diseases of N=916,619 patients, over the time span of $T = 5$ years (from 2012 to 2016). Initially, to obtain consistent results in the validation process, the diseases with a prevalence of less than 2% were obviated: only 47 diseases survived this screening. To enable the temporal evolution analysis, one sample per year was taken for each patient to obtain a vector containing categorical information about the presence or absence of each subsisted disease, in addition to age and gender. As a result, the dimension of these vectors is $d_0 = 49$.

The dataset was thereby organized in T temporal subsets in accordance with the time period. Each subset $\mathcal{Y}_o(t)$ was formed by N d_0-dimensional vectors $\mathbf{y}_o^n(t), n = 1, \dots, N$ containing patient's features, i.e. $\mathcal{Y}_o(t) \coloneqq \{\mathbf{y}_o^1(t), \mathbf{y}_o^2(t), \dots, \mathbf{y}_o^N(t)\}$, for $t = 1, \dots, T$. Let us denote the complete data set as $\boldsymbol{\mathcal{Y}}_o \coloneqq \{\mathcal{Y}_o(1), \mathcal{Y}_o(2), \dots, \mathcal{Y}_o(T)\}$.

Dimensionality Reduction by applying PCAmix

Since most of the selected features were categorical instead of quantitative, the dataset was a mixture of numerical and categorical variables. This dataset was processed by applying a mixture of the well-known Principal Component Analysis (PCA) and a Multiple Correspondence Analysis (MCA) to the database (the so called PCAmix algorithm) in order to obtain a new dataset, $\boldsymbol{\mathcal{Y}}$, whose feature space is reduced to the directions that provide a lower-error representation for the initial dataset $\boldsymbol{\mathcal{Y}}_o$. MCA can be envisaged as the counterpart of PCA for categorical data analysis and it allows the analysis of the pattern of relationships of several categorical dependent variables. To this end an indicator matrix is constructed, i.e. if the data table to be analyzed comprises N observations described by p categorical variables where each categorical variable has m_j levels and the sum of the m_j is equal to m, each level is coded as a binary variable in the N x m indicator matrix.

The PCAmix algorithm [1] reduces dimensions by applying a generalized singular value decomposition to a matrix composed of two sub-matrices $\mathbf{Z} = [\mathbf{Z}_1, \mathbf{Z}_2]$. Denoting the number of quantitative features as P, submatrix $\mathbf{Z}_1$, dimensioned $NT \times P$, is the standardized version of the quantitative features of the dataset $\boldsymbol{\mathcal{Y}}_o$ and the other submatrix $\mathbf{Z}_2$, dimensioned $NT \times (d_o - P)$, is the centered indicator matrix obtained from the categorical features. After formatting the dataset $\boldsymbol{\mathcal{Y}}_o$ as in $\mathbf{Z}$, a singular value decomposition is applied to this matrix, and a transformation matrix, $\mathbf{F}$, is computed to project the vectors in $\boldsymbol{\mathcal{Y}}_o$ to a d dimensional space $\boldsymbol{\mathcal{Y}}$, with $d < d_0$, by selecting the eigenvectors associated with the d largest singular values, also

denominated factor scores. The new dimension, d, was selected by applying the Karlis-Saporta-Spinaki rule [2], that is the criterion of retaining the factor scores significantly higher than the mean factor score. The transformed dataset is formed as $\mathcal{Y} := \{\mathcal{Y}(1), \mathcal{Y}(2), \dots, \mathcal{Y}(T)\}$ with $\mathcal{Y}(t) := \{\mathbf{y}^1(t), \mathbf{y}^2(t), \dots, \mathbf{y}^N(t)\}$, where each d-dimensional vector $\mathbf{y}^n(t)$ is obtained as

$$\mathbf{y}^n(t) = \mathbf{F}(\mathbf{y}_o^n(t) - \mathbf{m}_o); n = 1, \dots, N \quad (1)$$

where $\mathbf{m}_o$ is a d_0-dimension vector that guarantees that the distribution of the transformed database $\mathcal{Y}(t)$ is centered on zero, in all dimensions of the new observation space. When applying the Karlis-Saporta-Spinaki rule the reduced dimension was $d = 13$.

Initial soft clustering by applying FCM

It is assumed that the patient population is distributed into a set of clusters, corresponding to the different multimorbidity patterns. HMM are normally computed by applying an iterative algorithm named the Baum-Welch algorithm, as explained in the following subsection. In order to initialize some of the parameters used by the Baum Welch algorithm, the soft clustering procedure, Fuzzy C-means (FCM) algorithm is applied on the dataset $\mathcal{Y}$, to distribute the patients into a set of clusters. Each cluster is assumed to characterize a multimorbidity pattern. Cluster analysis involves assigning patients to clusters such that individuals in the same cluster are as similar as possible, while individuals belonging to different clusters are as dissimilar as possible. In our procedure the distribution of patients into the set of patterns is different in each of the years of study but it is assumed that the features characterizing each cluster through the corresponding centroid are stable over time. FCM is an unsupervised form of grouping in which each individual or patient can belong to more than one cluster, for that, it is assumed that each patient has some graded or fuzzy membership in each of the clusters. The FCM clustering process assigns a membership factor $u_{jn}(t)$ to each vector $\mathbf{y}_n(t)$, for $j = 1, \dots, K$ where K is the number of clusters. $u_{jn}(t)$ tells us the degree to which the nth patient in year t, belongs to the jth cluster. The similar/dissimilar properties are measured in FCM through a heuristic global cost function $J_m(\mathbf{U}, \mathbf{V}; \mathcal{Y})$, which is the weighted sum of squared errors within groups:

$$J_m(\mathbf{U}, \mathbf{V}; \mathcal{Y}) = \sum_{n=1}^{N} \sum_{t=1}^{T} \sum_{j=1}^{K} \left(u_{jn}(t)\right)^m \left\|\mathbf{y}_n(t) - \mathbf{v}_j\right\|^2 ; \quad 1 < m < \infty \qquad (2)$$

The norm $\left\|\mathbf{y}_n(t) - \mathbf{v}_j\right\|$ used in (2) defines a measure of similarity between a data point $\mathbf{y}_n(t)$ and the cluster prototypes $\mathbf{v}_j$ [3]. The weighting exponent parameter $m > 1$, is selected to adjust the blending of the different clusters. The objective function is iteratively minimized by assigning a membership $u_{jn}(t)$ to the vector $\mathbf{y}_n(t)$ in the jth cluster or pattern, and updating the cluster centroids $\mathbf{v}_j$ for $j = 1, \dots, K$. So, once we have applied FCM to the dataset $\mathcal{Y}$, we obtain a membership matrix $\mathbf{U} = \{u_{jn}(t)\}$, of size $NT \times K$ and a matrix of cluster centroids $\mathbf{V} = \{\mathbf{v}_1, \dots, \mathbf{v}_K\}$ of size $d \times K$. The membership variables and the centroids coordinates are computed as

$$u_{jn}(t) = \frac{1}{\sum_{i=1}^{K}\left(\frac{\left\|y_n(t)-v_j\right\|_A^2}{\left\|y_n(t)-v_i\right\|_A^2}\right)^{\frac{1}{m-1}}}; \quad 1 \leq j \leq K; \; 1 \leq n \leq N; 1 \leq t \leq T \qquad (3)$$

$$v_j = \frac{\sum_{n=1}^{N}\sum_{t=1}^{T}\left(u_{jn}(t)\right)^m y_n(t)}{\sum_{n=1}^{N}\sum_{t=1}^{T}\left(u_{jn}(t)\right)^m}; \quad 1 \leq j \leq K \qquad (4)$$

Since clustering algorithms are unsupervised machine-learning techniques, the model fitting to the dataset is traditionally computed through cost functions that depend on both the dataset and the clustering parameters, and are denoted as validation indexes. We computed three different well-known validation indexes to obtain the optimal number of clusters K and the optimal value of the fuzziness parameter *m*: the partition coefficient validation index (whose cost function is maximum for the optimal model), and the Xie-Beni and the partition entropy validation indexes whose cost functions are minimum for the optimal models [4]. We computed 100 runs. In each run the centroids in (4) have been randomly initialized, the FCM algorithm is applied and a final set of centroids is obtained. We checked m=1.1, 1.2 and 1.5 and K=5,...,40. The averaged performance obtained through the three validation indexes, resulted optimal for m=1.1 and K values between 6 and 12. Once this range was established, $K = 10$ clusters were identified by clinical assessment.

<u>HMM fitting by applying the Baum-Welch algorithm</u>

The observed data is assumed to be a time series of discrete time, for instance, the nth patient is represented by the observed time sequence $\boldsymbol{y}_n(t), t = 1, \ldots, T$. Therefore, to model the temporal evolution of patients through the different clusters or patterns, the sequential individual observations are assumed to follow a dynamic random process represented by a hidden Markov model (HMM). This means that each patient follows a longitudinal trajectory over $T = 5$ years $t_n := \{x_n(1), \ldots, x_n(T)\}$, through the clusters. For example, the nth patient could belong to cluster 1 between years 1 and 3 inclusive, and evolve into cluster 2 in the last two years. In this case, their longitudinal trajectory would be $t_n = \{1, 1, 1, 2, 2\}$. Some of the patients drop out from the database during the study follow-up, as 2.7% transfer out of the health system due to unknown causes, while 16.1% die before the last date of follow-up. To model this behavior, we have added two artificial clusters to the set of K patterns obtained from the FCM procedure application, one $(K+1)^{th}$ *cluster*, to model all people who move, from the first year they transfer out and another $(K+2)^{th}$ *cluster*, to model all people who die during the study time period, from the first year that they die. In the general case, each variable $x_n(t)$ can have any value among the $K+2$ possible states.

We adjust the observed time sequences, $\boldsymbol{y}_n(t), t = 1, \ldots, T, n = 1, \ldots, N$ to an HMM. In this process the longitudinal trajectory vector $t_n := \{x_n(1), \ldots, x_n(T)\}$ associated with the nth patient plays the role of a latent variable, as we do not have direct access to it, but we estimate it once all the parameters of the model have been identified. Each variable $x_n(t)$, for $t = 1, \ldots, T$ and $n = 1, \ldots, N$, have $K + 2$ possible values corresponding to the $K + 2$ clusters or patterns. Figure A1 shows a state transition diagram over time, where each row corresponds to one of the $K + 2$ states and each column corresponds to the latent variables $x_n(t)$. The red path shows the evolution of a patient through the longitudinal trajectory $x_n = \{1, 1, 1, 2, 2\}$.

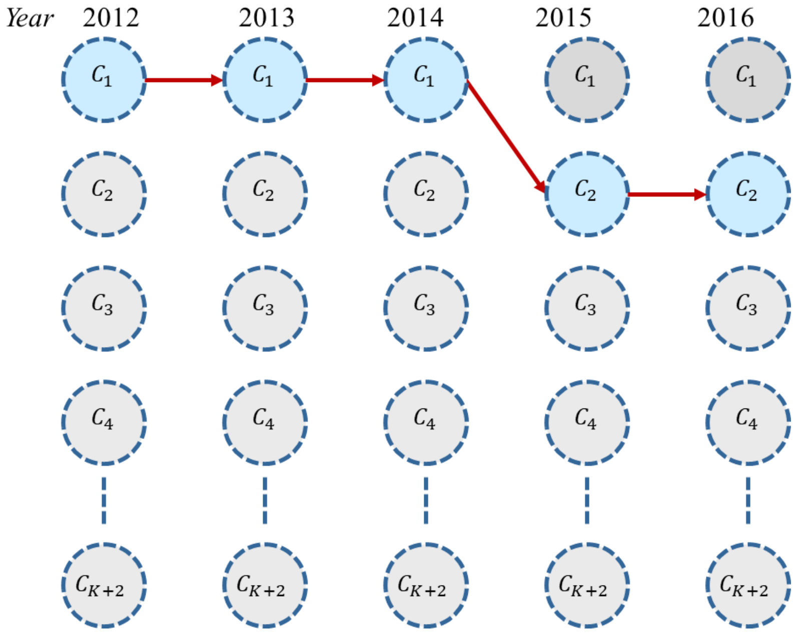

Figure A1. *State transition diagram of an HMM with $K + 2$ states. An individual trajectory $t_n = \{1, 1, 1, 2, 2\}$ is shown by red arrows.*

A joint longitudinal representation of the latent variables associated with the nth patient and the observed sequence is shown in Figure A2 to emphasize the fact that each observed vector $\boldsymbol{y}_n(t)$ is conditioned on the state of the corresponding latent variable $x_n(t)$.

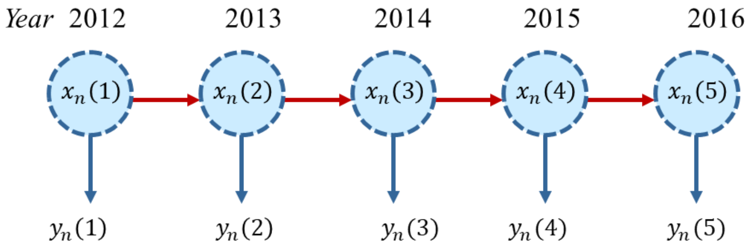

Figure A2. *Joint longitudinal representation of the latent variables $x_n(t)$, associated with the nth patient and the observed sequence $\boldsymbol{y}_n(t)$.*

In the following, the full set of latent variables is denoted as $\boldsymbol{X}$, i.e. $\boldsymbol{X} := \{x_n(t); n = 1, \dots, N, t = 1, \dots, T\}$.

The conditional distribution of the observed variables $\boldsymbol{y}_n(t)$, are known as emission probabilities and they are modeled as a Gaussian distributed over a d −dimensional space in this application, i.e. the conditioned probability density function $f(y_n(t)|x_n(t) = j) : \mathcal{N}(\boldsymbol{m}_j, \boldsymbol{C}_j)$ for $j = 1, \dots, K$, where $\boldsymbol{m}_j$ is the mean vector and $\boldsymbol{C}_j$ is the covariance matrix associated with the hidden state $x_n(t) = j$. The observations for the $K+1$ and $K+2$ patterns do not need to be modeled, since on the one hand there are no registered features in these cases, and on the other, these states are known in advance. Even so, in what follows this distinction will not always be made in order not to excessively load the mathematical formulas.

A first order HMM model is fully characterized by the set of parameters defined as $\boldsymbol{\theta} := \{\pi_j, p_{ij}, \boldsymbol{m}_j, \boldsymbol{C}_j, i, j = 1, \dots, K+2\}$, where π_j are the initial state probabilities and p_{ij} are the transition probabilities defined as $p_{ij} = \Pr\{x_n(t) = i | x_n(t-1) = j\}$, i.e. p_{ij} is the probability that any patient jumps from the jth group to the ith group in a year's time.

Once we have defined the model, our main challenge is to estimate the model parameters $\boldsymbol{\theta}$. Given the observed dataset $\boldsymbol{\mathcal{Y}}$, the maximum likelihood procedure consists of seeking the model parameters that most likely have generated $\boldsymbol{\mathcal{Y}}$. To this end, adopting the independent and identically distributed random variables assumption, whereby each patient sequence is independent of each other, the likelihood of the joint probability distribution of the complete dataset $\{\boldsymbol{\mathcal{Y}}, \boldsymbol{X}\}$ denoted as $p(\boldsymbol{\mathcal{Y}}, \boldsymbol{X}; \boldsymbol{\theta})$, has to be maximized in terms of $\boldsymbol{\theta}$. As a closed solution for the parameters does not exist, this problem is traditionally solved by applying the Expectation-Maximization (EM) algorithm, also called the Baum-Welch algorithm in the context of HMM [5,6]. The BW algorithm is well documented in the literature. It is a procedure that iteratively alternates between the expectation step (E-Step) and the maximization step (M-Step). It must be initialized by choosing starting values $\boldsymbol{\theta}^o$ for the model parameters. In our work we have randomly initialized the transition probabilities $\{p_{ij}^o, i, j = 1, \dots, K+2\}$, respecting the summation constraints associated with their probabilistic nature. Further, the parameters of the emission distribution $\{\boldsymbol{m}_j, j = 1, \dots, K\}$, have been initialized as the centroids obtained by applying the FCM clustering algorithm given in (4), $\boldsymbol{m}_j^o = \boldsymbol{v}_j$, and the initial associated covariance matrices have been taken as:

$$\boldsymbol{C}_j^o = diag(c_1, \dots, c_K); \quad \{c_1, \dots, c_K\} = 0.1; \quad 1 \leq j \leq K \qquad (5)$$

Initial state probabilities $\{\pi_j^0; j = 1, \dots, K+2\}$ have been taken as the probability of being in a certain cluster for the previous FCM clustering. To compute these parameters, all the memberships have been summed per cluster divided by the total number of individuals.

The BW algorithm alternatively iterates between an expectation step and a maximization step. In the expectation step, we compute the expectation of the log likelihood function $\log p(\boldsymbol{\mathcal{Y}}, \boldsymbol{X}; \boldsymbol{\theta})$ with respect to the latent variables conditioned to the observed sequences and

given a previous estimate of the set of HMM parameters. In the maximization step the conditional expectation obtained in the expectation step is maximized with respect to the set of parameters. Once the values of the log-likelihood function have converged, the algorithm is stopped and it delivers the final set of model parameters $\boldsymbol{\theta}^f := \{\pi_j^f, p_{ij}^f, \mathbf{m}_j^f, \mathbf{C}_j^f, i,j = 1,\dots,K+2\}$.

Trajectory decoding

Once the final parameters are available, the longitudinal trajectories $\{t_n; n = 1,\dots,N\}$ followed by the individuals can be inferred. In this process, given an observed sequence $\mathbf{y}_n(t), t = 1,\dots,T$ and the set of final parameters $\boldsymbol{\theta}^f$, the best longitudinal trajectory $\hat{t}_n := \{\hat{x}_n(1),\dots,\hat{x}_n(T)\}$ is computed by maximizing the probability of the observed sequence conditioned to the set of final parameters. Note, that we distinguish between the latent variable $x_n(t)$, and the value $\hat{x}_n(t)$, that is estimated for this variable. This problem is efficiently solved by applying the well-known Viterbi algorithm and it is repeated N times, one for each patient. Further details of the Viterbi algorithm are shown in [7] and [8]. As a result of this decoding process, the N longitudinal trajectories are the entries of the matrix $\widehat{\mathbf{X}}$, defined as $\widehat{\mathbf{X}} := [\hat{t}_1; \hat{t}_2; \dots; \hat{t}_N]$, and in such a way that each of the N rows corresponds to one of the individual trajectories.

Expected and decoded values

After obtaining the parameters of the best model with the Baum-Welch algorithm and having decoded the observations with Viterbi, a comparison between expected values (Baum-Welch model) and decoded values (Viterbi algorithm) was conducted, showing a good agreement between these values.

	Transition probabilities from Baum-Welch model - Transition probabilities from Viterbi decoding											
Origin \ Dest	C1 - Non-Specific	C2 - Eye Impairment and Mental	C3 - Minority Metabolic Autoimmune-Inflammatory	C4 - Cardio-Circulatory and Renal	C5 - Cardio-Circulatory, Mental, Respiratory and Genitourinary	C6 - Nervous, Digestive and Circulatory	C7 - Respiratory and Ear	C8 - Digestive	C9 - Nervous, Musculoskeletal and Minor	C10 - Multisystem Pattern	Dropout	Death
C1 - Non-Specific	64.6% - 66.1%	6.4% - 3.5%	4% - 4.2%	1.4% - 1.7%	1.6% - 2%	1.9% - 2.6%	1% - 1.1%	0.6% - 1.2%	0.3% - 0.5%	0.1% - 0.1%	3.2% - 3.2%	15% - 13.7%
C2 - Eye Impairment and Mental	1.3% - 0.3%	74.7% - 79%	2.6% - 0.7%	2.9% - 2%	1.4% - 0.7%	1.7% - 1.9%	1.2% - 1%	1% - 0.9%	2.8% - 2.1%	0.8% - 1.5%	1.9% - 2%	7.7% - 7.9%
C3 - Minority Metabolic Autoimmune-Inflammatory	2.4% - 1.3%	1.3% - 0.1%	70.2% - 76.7%	0.8% - 0.6%	1.1% - 0.3%	1.2% - 1.1%	0.7% - 0.5%	1.3% - 0.7%	0.7% - 0.1%	0.3% - 0.3%	2.1% - 2.1%	17.8% - 16.1%
C4 - Cardio-Circulatory and Renal	0.3% - 0.1%	0.3% - 0%	0.2% - 0%	55.4% - 59.2%	0.5% - 0%	0.4% - 0.3%	0.2% - 0.1%	0.6% - 0.2%	0.1% - 0%	0.2% - 0.1%	2.8% - 2.9%	38.9% - 37.1%
C5 - Cardio-Circulatory, Mental, Respiratory and Genitourinary	0.6% - 0.2%	0.3% - 0%	0.6% - 0.1%	0.5% - 0%	60.5% - 67.3%	0.9% - 0.8%	0.2% - 0%	0.9% - 0.5%	0% - 0%	0% - 0%	2.5% - 2.3%	32.8% - 28.8%
C6 - Nervous, Digestive and Circulatory	1.4% - 1.5%	0.8% - 0.5%	0.4% - 0.2%	0.2% - 0.1%	0.3% - 0.2%	58.4% - 61.2%	0.2% - 0.2%	0.2% - 0.1%	0.6% - 0.1%	0.2% - 0.1%	4% - 4%	33.4% - 31.8%
C7 - Respiratory and Ear	1.9% - 1.7%	1.8% - 1.4%	0.6% - 0.4%	0.5% - 0.5%	0.4% - 0.3%	0.6% - 0.7%	76.7% - 77.9%	0.2% - 0.2%	0.5% - 0.4%	0.6% - 0.9%	2% - 2.1%	14.2% - 13.7%
C8 - Digestive	1.3% - 2.2%	2.1% - 1.4%	0.9% - 0.5%	0.4% - 0.3%	0.3% - 0.3%	0.5% - 0.5%	0.2% - 0.2%	65.4% - 68.4%	0.2% - 0.1%	0.7% - 0.4%	2.5% - 2.5%	25.4% - 23.3%
C9 - Nervous, Musculoskeletal and Minor	0.4% - 0.3%	3% - 0.7%	0.8% - 0.1%	0.7% - 0.4%	0.1% - 0%	1.9% - 0.5%	0.7% - 0.3%	0.3% - 0.1%	84.4% - 92.1%	2.3% - 1%	1.2% - 1.1%	4.3% - 3.3%
C10 - Multisystem Pattern	0.3% - 0.1%	1.7% - 0.4%	0.3% - 0%	0.4% - 0.1%	0% - 0%	0.6% - 0.1%	0.6% - 0.2%	0.4% - 0%	0.7% - 0%	81.4% - 85.4%	1.9% - 1.9%	11.7% - 11.6%
Dropout	0% - 0%	0% - 0%	0% - 0%	0% - 0%	0% - 0%	0% - 0%	0% - 0%	0% - 0%	0% - 0%	0% - 0%	100% - 100%	0% - 0%
Death	0% - 0%	0% - 0%	0% - 0%	0% - 0%	0% - 0%	0% - 0%	0% - 0%	0% - 0%	0% - 0%	0% - 0%	0% - 0%	100% - 100%

	Initial probabilities from Baum-Welch model - Initial probabilities from Viterbi decoding											
Clusters	C1 - Non-Specific	C2 - Eye Impairment and Mental	C3 - Minority Metabolic Autoimmune-Inflammatory	C4 - Cardio-Circulatory and Renal	C5 - Cardio-Circulatory, Mental, Respiratory and Genitourinary	C6 - Nervous, Digestive and Circulatory	C7 - Respiratory and Ear	C8 - Digestive	C9 - Nervous, Musculoskeletal and Minor	C10 - Multisystem Pattern	Dropout	Death
Initial Probabilities	48.1% - 42%	23% - 19.3%	6.8% - 7.9%	4.6% - 6.6%	3.7% - 5.9%	3.7% - 4.6%	4.5% - 4.5%	3% - 3.9%	2.1% - 3.6%	0.5% - 7.9%	0% - 0%	0% - 0%

Table A1. Transition and initial probabilities from the Baum-Welch algorithm and Viterbi decoding

Validation procedure

Both, the FCM algorithm and the BW algorithm are iterative procedures that can converge to a local maximum, so a validation technique has to be applied to substantiate the optimality of the solution. The initialization of the set of parameters can influence the quality of results. To obtain the model that best fits the data, both the FCM initial clustering, using the value of K clusters previously validated by applying FCM validation indexes, and the Baum-Welch algorithm are computed many times (100 runs) with different random initial parameters $\boldsymbol{\theta}^{\boldsymbol{o}}$ or priors, while keeping track of the log-likelihood of the data conditioned to the delivered set of final parameters, i.e. $\log p(\mathcal{Y}, \widehat{\boldsymbol{X}}; \boldsymbol{\theta}^f)$. The best model is selected as the one that produces the highest validation score $\log p(\mathcal{Y}, \widehat{\boldsymbol{X}}; \boldsymbol{\theta}^f)$.

Once the best model has been selected, the log-likelihood function of the observation $\log p(\mathcal{Y}; \boldsymbol{\theta}^f)$ is compared with the theoretical log-likelihood function that would result from a randomly generated dataset $\boldsymbol{W}$ of the same size $N = 916{,}619$ and distributed as $p(\boldsymbol{W}; \boldsymbol{\theta}^f)$. Given that N is large, the log-likelihood function does not depart much from the theoretical value if the model is correct. On the other hand, when there is a misalignment between the model $\boldsymbol{\theta}$ that generates the observed data and the parameters $\boldsymbol{\theta}^f$ used to compute the log-likelihood function, the measured $\log p(\boldsymbol{W}; \boldsymbol{\theta}^f)$ function decreases. To show this effect, the loss in the value of the log-likelihood function has been measured with respect to the parameter's misalignment, modeled by independent random variables $\Delta_i; i = 1, \ldots, card(\boldsymbol{\theta})$ as shown in Figure A3. The result of 100 independent runs is shown, i.e. 100 different synthetic datasets $\boldsymbol{W}$ were generated. With each dataset $\boldsymbol{W}$**,** the log-likelihood function $\log p(\boldsymbol{W}; \widehat{\boldsymbol{\theta}})$ was computed, generating noisy parameters by means of adding zero mean random noise $(-1 \leq \Delta_i \leq +1)$ to each parameter. Each individual parameter θ_i has been replaced by a noisy counterpart $\hat{\theta}_i = \theta_i(1 + \alpha\Delta_i)$ where α goes from 0 to 1 and represents the maximum misalignment level in terms of the corrupted parameter θ_i.

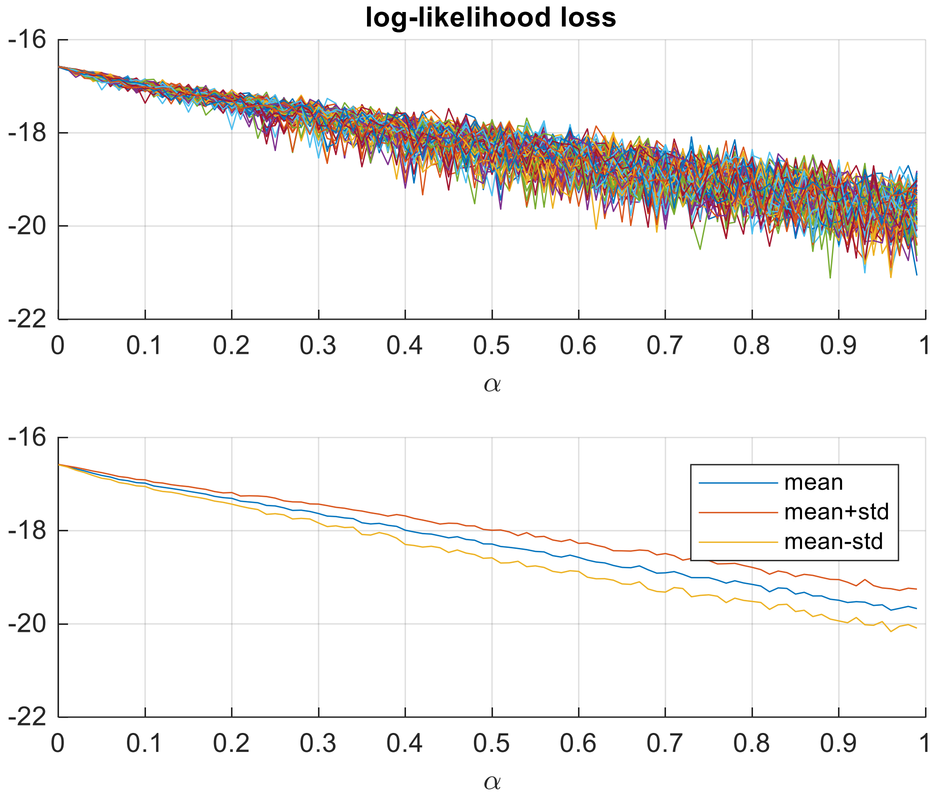

Figure A3. Loss of the log-likelihood function $\log p(\boldsymbol{W};\widehat{\boldsymbol{\theta}})$ versus the parameter misalignment level α. In the upper figure we show the log-likelihood loss of the 100 independent runs and in the lower one the mean, mean plus and mean minus standard deviation are given.

Obviously, a mismatch in the model results in the reduction of the likelihood function. This could be used as a method to validate the model assumed in the HMM. To that end, we generated Gaussian synthetic data assuming the parameters obtained with the HMM model. The log-likelihood function of the observation $\log p(\boldsymbol{\mathcal{Y}};\boldsymbol{\theta}^f)$ was -16.4, which is in high agreement with the computed model $\boldsymbol{\theta}^f$ and correctly fitted the observed data $\boldsymbol{\mathcal{Y}}$.

References

1. Chavent M, Kuentz-Simonet V, Labenne A, Saracco J. Multivariate Analysis of Mixed Data: The R Package PCAmixdata. arXiv:1411.4911v4 [stat.CO]. Cornell University; 2017.

2. Karlis D, Saporta G, Spinakis A. A simple rule for the selection of Principals components. Communications in Statistics - Theory and Methods. 2003; 32:3, 643-666,

3. Ramze Rezaee M, Lelieceldt BPF, Reiber JHC. A new cluster validity index for the fuzzy c-mean.Pattern Recognition Letters. 1998; 19, 237-246.

4. Zhao Q. Cluster validity in Clustering Methods. Thesis, The University of Eastern Finland. Available from: http://cs.joensuu.fi/sipu/pub/qinpei-thesis.pdf

5. Rabiner LR. A tutorial on hidden Markov models and selected applications in speech recognition. Proc. IEEE. 1989; 77:257–86.

6. Barber D. Bayesian reasoning and Machine Learning. Cambridge: Cambridge University Press; 2012.

7. Bishop CM. Pattern Recognition and Machine Learning (Information Science and Statistics). Singapore: Springer; 2006.

8. Theodoridis S. Machine Learning. A Bayesian and Optimization Perspective. London: Elsevier; 2015.

Supplementary 3. Multimorbidity patterns with Hidden Markov Models at baseline (year 2012) and final year (2016) of the study

CLUSTER 1 - NON-SPECIFIC PATTERN

N_{2012} = 384787 (42.0% , 52.5% female) N_{2016} = 258700 (34.8%, 53.3% female)

Disease	Prevalence		O/E ratio		Exclusivity	
	2012	2016	2012	2016	2012	2016
Prostate diseases	16.28	20.17	0.97	1.00	40.77	34.89
Hypertension	60.31	66.72	0.85	0.87	35.65	30.34
Solid neoplasms	12.60	15.78	0.84	0.83	35.37	28.97
Dyslipidemia	41.72	47.00	0.82	0.85	34.41	29.43
Dementia	5.01	5.37	0.78	0.71	32.54	24.85
Osteoporosis	12.34	12.51	0.75	0.74	31.28	25.84
Osteoarthritis and other degenerative joint diseases	23.72	30.07	0.72	0.75	30.34	25.96
Other musculoskeletal and joint diseases	9.31	13.03	0.68	0.71	28.70	24.58
Venous and lymphatic diseases	9.46	12.85	0.68	0.71	28.65	24.79
Deafness, hearing impairment	6.59	10.72	0.67	0.71	28.11	24.59
Other psychiatric and behavioral diseases	3.34	5.00	0.65	0.60	27.41	20.82
Dorsopathies	8.83	13.21	0.65	0.68	27.26	23.65
Cerebrovascular disease	5.57	5.28	0.64	0.61	26.70	21.37
Obesity	18.15	22.44	0.63	0.68	26.57	23.51

CLUSTER 2 - EYE IMPAIRMENT AND MENTAL DISEASES

N_{2012} = 177087 (19.3%, 73.7% female) N_{2016} = 155088 (20.8%, 73.3% female)

Disease	Prevalence		O/E ratio		Exclusivity	
	2012	2016	2012	2016	2012	2016
Glaucoma	18.90	21.83	**2.62**	**2.34**	50.59	48.75
Other eye diseases	16.64	20.94	**2.21**	1.98	42.74	41.34
Neurotic, stress-related and somatoform diseases	28.96	33.27	**2.15**	1.82	41.56	37.94
Depression and mood diseases	31.37	33.59	1.93	1.71	37.35	35.54
Migraine and facial pain syndromes	4.62	4.85	1.63	1.42	31.47	29.58
Diabetes	39.92	41.01	1.59	1.52	30.67	31.66
Thyroid diseases	14.34	17.30	1.49	1.35	28.72	28.05
Obesity	42.02	43.46	1.47	1.31	28.31	27.28
Dyslipidemia	67.55	69.31	1.33	1.25	25.64	26.01
Cataract and other lens diseases	22.64	31.51	1.32	1.24	25.59	25.88
Other genitourinary diseases	12.07	14.76	1.29	1.15	25.00	23.89
Allergy	5.57	8.87	1.26	1.14	24.40	23.69
Sleep disorders	12.12	21.10	1.25	1.15	24.18	23.87
Ear, nose, throat diseases	5.07	6.95	1.20	1.08	23.15	22.46

COPD, emphysema, chronic bronchitis	7.37	8.28	0.62	0.61	25.88	21.09

CLUSTER 3 - MINORITY METABOLIC AUTOIMMUNE-INFLAMATORY PATTERN

N_{2012} = 72190 (7.9%, 43.5% female) N_{2016} = 73237 (9.8%, 43.0% female)

Disease	Prevalence		O/E ratio		Exclusivity	
	2012	2016	2012	2016	2012	2016
Other metabolic diseases	15.58	16.58	**7.52**	**5.70**	59.21	56.11
Inflammatory arthropathies	49.21	53.01	**7.22**	**5.41**	56.88	53.25
Autoimmune diseases	30.75	31.23	**7.16**	**5.46**	56.41	53.76
Chronic kidney diseases	28.99	39.36	1.73	1.49	13.61	14.64
Anemia	28.09	31.09	1.54	1.36	12.10	13.37
Prostate diseases	25.02	29.87	1.49	1.49	11.76	14.63
Solid neoplasms	21.69	25.94	1.45	1.37	11.43	13.48
Osteoporosis	22.38	21.08	1.35	1.25	10.64	12.32
Esophagus, stomach and duodenum diseases	11.58	17.16	1.33	1.19	10.44	11.70
Thyroid diseases	12.20	14.55	1.26	1.13	9.96	11.15
Peripheral vascular disease	3.85	4.89	1.15	1.11	9.05	10.94
Other musculoskeletal and joint diseases	15.52	19.61	1.14	1.06	8.98	10.47
Osteoarthritis and other degenerative joint diseases	37.27	43.00	1.14	1.07	8.94	10.51
COPD, emphysema, chronic bronchitis	13.45	15.11	1.13	1.11	8.86	10.89

Hypertension	82.04	84.80	1.16	1.11	22.32	23.11

CLUSTER 4 - CARDIO-CIRCULATORY AND RENAL PATTERN

N_{2012} = 60192 (6.6%, 70.8% female) N_{2016} = 46244 (6.2%, 70.9% female)

Disease	Prevalence		O/E ratio		Exclusivity	
	2012	2016	2012	2016	2012	2016
Cardiac valve diseases	43.18	53.79	**7.60**	**6.88**	49.89	42.77
Heart failure	57.79	65.65	**7.15**	**6.55**	46.95	40.73
Atrial fibrillation	54.10	62.24	**6.18**	**5.41**	40.58	33.63
Bradycardias and conduction diseases	14.76	19.46	**5.31**	**4.29**	34.88	26.65
Ischemic heart disease	34.68	32.84	**3.33**	**3.02**	21.87	18.78
Chronic kidney diseases	41.91	54.27	**2.50**	**2.05**	16.41	12.75
Cerebrovascular disease	20.44	18.17	**2.33**	**2.11**	15.33	13.15
Anemia	41.91	44.98	**2.29**	1.97	15.05	12.22
Other eye diseases	15.43	18.07	**2.05**	1.71	13.48	10.65
Thyroid diseases	17.42	21.27	1.81	1.66	11.85	10.29
Diabetes	42.27	39.96	1.68	1.48	11.04	9.20
Dementia	10.65	10.86	1.65	1.44	10.82	8.98
Chronic pancreas, biliary tract and gallbladder diseases	4.71	6.07	1.58	1.38	10.38	8.59
Peripheral vascular disease	5.23	5.94	1.56	1.35	10.27	8.40

Dorsopathies	15.09	19.71	1.11	1.02	8.74	9.99

Glaucoma	11.06	12.64	1.53	1.35	10.07	8.42

CLUSTER 5 - CARDIO-CIRCULATORY MENTAL, RESPIRATORY AND GENITOURINARY PATTERN

N_{2012} = 54324 (5.9%, 6.7% female) N_{2016} = 45463 (6.1%, 6.4% female)

Disease	Prevalence		O/E ratio		Exclusivity	
	2012	2016	2012	2016	2012	2016
Peripheral vascular disease	29.36	32.92	**8.77**	**7.49**	51.99	45.77
Other psychiatric and behavioral diseases	24.73	29.95	**4.84**	**3.59**	28.68	21.92
COPD, emphysema, chronic bronchitis	44.07	45.11	**3.69**	**3.30**	21.84	20.19
Ischemic heart disease	36.59	37.91	**3.51**	**3.49**	20.83	21.31
Prostate diseases	57.34	64.72	**3.42**	**3.22**	20.28	19.68
Bradycardias and conduction diseases	8.35	12.77	**3.00**	**2.81**	17.81	17.19
Cerebrovascular disease	21.74	20.20	**2.48**	**2.35**	14.72	14.37
Heart failure	17.58	21.86	**2.18**	**2.18**	12.89	13.33
Peripheral neuropathy	10.67	14.25	1.99	1.79	11.80	10.96
Dementia	12.62	13.86	1.95	1.84	11.58	11.26
Ear, nose, throat diseases	8.01	11.08	1.89	1.72	11.23	10.50
Deafness, hearing impairment	18.58	25.54	1.89	1.68	11.18	10.29
Atrial fibrillation	15.81	21.28	1.81	1.85	10.70	11.31
Diabetes	44.66	45.51	1.78	1.69	10.53	10.30

CLUSTER 6 - NERVOUS, DIGESTIVE AND CIRCULATORY PATTERN

N_{2012} = 42422 (4.6%, 55.6% female) N_{2016} = 41120 (5.5%, 55.0% female)

Disease	Prevalence		O/E ratio		Exclusivity	
	2012	2016	2012	2016	2012	2016
Parkinson and parkinsonism	45.67	44.73	**20.09**	**16.03**	92.99	88.60
Other neurological diseases	48.97	56.71	**15.73**	**11.92**	72.79	65.91
Other digestive diseases	10.77	11.01	**4.48**	**3.44**	20.74	19.01
Dementia	25.71	23.39	**3.98**	**3.11**	18.42	17.20
Cerebrovascular disease	23.56	19.77	**2.69**	**2.30**	12.45	12.72
Colitis and related diseases	27.16	23.70	1.89	1.46	8.76	8.08
Depression and mood diseases	26.00	28.55	1.60	1.45	7.42	8.01
Anemia	27.37	30.54	1.50	1.33	6.93	7.38
Sleep disorders	12.85	22.72	1.33	1.23	6.14	6.82
Prostate diseases	21.80	25.37	1.30	1.26	6.02	6.98
Neurotic, stress-related and somatoform diseases	16.41	19.68	1.22	1.08	5.64	5.95
Chronic kidney diseases	19.22	27.80	1.15	1.05	5.30	5.81
Bradycardias and conduction diseases	3.14	4.71	1.13	1.04	5.22	5.74
Atrial fibrillation	9.75	11.72	1.11	1.02	5.16	5.63

Other eye diseases	13.24	17.30	1.76	1.64	10.43	10.02

Cataract and other lens diseases	18.76	25.25	1.10	0.99	5.08	5.50

CLUSTER 7 - RESPIRATORY AND EAR PATTERN

N_{2012} = 41644 (4.5%, 74.8% female) N_{2016} = 38961 (5.2%, 74.0% female)

Disease	Prevalence		O/E ratio		Exclusivity	
	2012	2016	2012	2016	2012	2016
Asthma	87.37	86.44	**18.34**	**15.71**	83.33	82.29
Allergy	16.48	23.65	**3.74**	**3.03**	16.99	15.88
COPD, emphysema, chronic bronchitis	40.94	43.59	**3.42**	**3.19**	15.55	16.72
Ear, nose, throat diseases	9.14	11.98	**2.16**	1.86	9.83	9.73
Osteoporosis	25.83	24.50	1.56	1.45	7.08	7.62
Venous and lymphatic diseases	20.95	25.01	1.51	1.39	6.87	7.27
Obesity	42.28	46.25	1.47	1.39	6.70	7.30
Esophagus, stomach and duodenum diseases	11.80	18.63	1.35	1.29	6.14	6.75
Thyroid diseases	12.70	16.07	1.32	1.25	5.98	6.55
Sleep disorders	12.55	22.11	1.30	1.20	5.89	6.29
Osteoarthritis and other degenerative joint diseases	41.86	48.40	1.28	1.20	5.79	6.29
Depression and mood diseases	20.45	23.82	1.26	1.21	5.73	6.34
Neurotic, stress-related and somatoform diseases	16.54	21.24	1.23	1.16	5.58	6.09
Heart failure	9.80	11.98	1.21	1.20	5.51	6.26

CLUSTER 8 - DIGESTIVE PATTERN

N_{2012} = 35692 (3.9%, 55.8% female) N_{2016} = 31744 (4.3%, 56.2% female)

Disease	Prevalence		O/E ratio		Exclusivity	
	2012	2016	2012	2016	2012	2016
Chronic liver diseases	58.28	50.84	**23.60**	**21.29**	91.91	90.87
Chronic pancreas, biliary tract and gallbladder diseases	33.35	40.84	**11.19**	**9.30**	43.57	39.69
Other digestive diseases	17.44	19.88	**7.26**	**6.21**	28.26	26.50
Esophagus, stomach and duodenum diseases	17.79	24.88	**2.04**	1.72	7.93	7.35
Other metabolic diseases	3.93	5.12	1.90	1.76	7.38	7.50
Solid neoplasms	24.46	30.00	1.64	1.58	6.37	6.76
Other genitourinary diseases	14.06	18.44	1.51	1.43	5.87	6.12
Anemia	27.34	33.34	1.50	1.46	5.82	6.22
Other psychiatric and behavioral diseases	7.29	11.05	1.43	1.32	5.55	5.64
Peripheral vascular disease	4.31	5.62	1.29	1.28	5.01	5.46
Diabetes	32.33	33.69	1.29	1.25	5.01	5.33
Colitis and related diseases	18.31	20.08	1.28	1.24	4.97	5.28
COPD, emphysema, chronic bronchitis	14.77	16.08	1.24	1.18	4.81	5.02
Thyroid diseases	11.59	14.82	1.20	1.15	4.68	4.92

Other genitourinary diseases	11.03	14.16	1.18	1.10	5.37	5.76

Autoimmune diseases	5.04	5.72	1.17	1.00	4.57	4.27

CLUSTER 9 - NERVOUS, MUSCULOSKELETAL, AND MINOR DISEASES PATTERN

N_{2012} = 33283 (3.6%, 92.6% female) N_{2016} = 36522 (4.9%, 92.3% female)

Disease	Prevalence		O/E ratio		Exclusivity	
	2012	2016	2012	2016	2012	2016
Peripheral neuropathy	44.00	48.08	**8.21**	**6.05**	29.81	29.71
Dorsopathies	60.18	66.21	**4.43**	**3.41**	16.08	16.74
Other musculoskeletal and joint diseases	57.04	63.04	**4.19**	**3.42**	15.22	16.79
Other genitourinary diseases	28.92	33.40	**3.10**	**2.60**	11.26	12.74
Venous and lymphatic diseases	40.63	45.32	**2.93**	**2.51**	10.65	12.34
Migraine and facial pain síndromes	8.03	8.49	**2.83**	**2.49**	10.28	12.20
Other neurological diseases	8.60	12.09	**2.76**	**2.54**	10.03	12.48
Other digestive diseases	5.73	6.10	**2.38**	1.90	8.66	9.35
Osteoarthritis and other degenerative joint diseases	73.88	79.76	**2.25**	1.98	8.17	9.72
Osteoporosis	35.44	32.07	**2.14**	1.90	7.77	9.35
Depression and mood diseases	32.61	35.97	**2.01**	1.83	7.30	8.97
Neurotic, stress-related and somatoform diseases	26.14	31.56	1.94	1.73	7.05	8.48
Obesity	52.27	55.09	1.82	1.66	6.62	8.15
Other eye diseases	12.61	15.53	1.68	1.47	6.09	7.23

CLUSTER 10 - MULTISYSTEM PATTERN

N_{2012} = 14998 (1.6%, 89.9% female) N_{2016} = 16828 (2.3%, 89.2% female)

Disease	Prevalence		O/E ratio		Exclusivity	
	2012	2016	2012	2016	2012	2016
Migraine and facial pain syndromes	19.98	20.99	**7.05**	**6.15**	11.53	13.90
Other digestive diseases	16.63	18.91	**6.92**	**5.91**	11.33	13.36
Ear, nose, throat diseases	20.42	25.15	**4.83**	**3.90**	7.90	8.83
Allergy	20.72	27.63	**4.70**	**3.54**	7.69	8.01
Neurotic, stress-related and somatoform diseases	58.46	62.85	**4.34**	**3.44**	7.11	7.78
Esophagus, stomach and duodenum diseases	37.16	45.42	**4.26**	**3.14**	6.96	7.11
Chronic pancreas, biliary tract and gallbladder diseases	11.83	13.67	**3.97**	**3.11**	6.49	7.04
Depression and mood diseases	62.64	65.35	**3.86**	**3.32**	6.32	7.51
Colitis and related diseases	52.50	55.38	**3.66**	**3.41**	5.99	7.72
Other eye diseases	27.31	29.71	**3.63**	**2.81**	5.94	6.37
Other genitourinary diseases	32.58	37.32	**3.49**	**2.90**	5.72	6.56
Sleep disorders	32.55	45.61	**3.36**	**2.48**	5.50	5.60
Other metabolic diseases	6.79	8.55	**3.28**	**2.94**	5.36	6.65
Osteoporosis	51.69	48.73	**3.12**	**2.89**	5.11	6.55

Ear, nose, throat diseases	7.08	9.83	1.68	1.52	6.08	7.49

Deafness, hearing impairment	30.33	36.62	**3.08**	**2.41**	5.04	5.46
Asthma	13.66	14.15	**2.87**	**2.57**	4.69	5.82
Dorsopathies	37.21	42.66	**2.74**	**2.20**	4.48	4.97
Autoimmune diseases	11.74	13.66	**2.74**	**2.39**	4.48	5.40
Glaucoma	19.10	21.71	**2.65**	**2.33**	4.33	5.26
Cataract and other lens diseases	42.73	50.26	**2.50**	1.98	4.09	4.48
Peripheral neuropathy	13.26	15.96	**2.47**	**2.01**	4.05	4.54
Chronic liver diseases	6.03	5.42	**2.44**	**2.27**	4.00	5.13
Venous and lymphatic diseases	33.10	36.61	**2.39**	**2.03**	3.91	4.59
Thyroid diseases	22.48	26.65	**2.33**	**2.07**	3.81	4.69
Other musculoskeletal and joint diseases	30.06	34.75	**2.21**	1.89	3.61	4.26
Other neurological diseases	6.87	9.47	**2.21**	1.99	3.61	4.51
Dementia	14.21	16.87	**2.20**	**2.24**	3.60	5.08
Cardiac valve diseases	11.79	14.81	**2.07**	1.89	3.39	4.29

Supplementary 4. Variables characterizing each cluster in the study at baseline and at the end of the study (N = 916,619)

Variables	Cluster 1 - Non-Specific	Cluster 2 - Eye Impairment and Mental	Cluster 3 - Minority Metabolic Autoimmune-Inflammatory	Cluster 4 - Cardio-Circulatory and Renal	Cluster 5 - Cardio-Circulatory, Mental, Respiratory and Genitourinary	Cluster 6 - Nervous, Digestive and Circulatory	Cluster 7 - Respiratory and Ear	Cluster 8 - Digestive	Cluster 9 - Nervous, Musculoskeletal and Minor	Cluster 10 - Multisystem Pattern	Overall (Living people)	
	N_{2012}=384787 (42.0%) - N_{2016}=258700 (34.8%)	N_{2012}=177087 (19.3%) - N_{2016}=155008 (20.8%)	N_{2012}=72190 (7.9%) - N_{2016}=73237 (9.8%)	N_{2012}=60192 (6.6%) - N_{2016}=46244 (6.2%)	N_{2012}=54324 (5.9%) - N_{2016}=45463 (6.1%)	N_{2012}=42422 (4.6%) - N_{2016}=41120 (5.5%)	N_{2012}=41644 (4.5%) - N_{2016}=38961 (5.2%)	N_{2012}=35692 (3.9%) - N_{2016}=31744 (4.3%)	N_{2012}=33283 (3.6%) - N_{2016}=36522 (4.9%)	N_{2012}=14998 (1.6%) - N_{2016}=16828 (2.3%)	N_{2012}=916619 - N_{2016}= 743827	N_{2012} - N_{2016}
**Sex:**												916619 - 743827
Female	201958 (52.5%) - 137931 (53.3%)	130572 (73.7%) - 113574 (73.3%)	31395 (43.5%) - 31490 (43.0%)	42638 (70.8%) - 32786 (70.9%)	3618 (6.7%) - 2902 (6.4%)	23577 (55.6%) - 22601 (55.0%)	31143 (74.8%) - 28838 (74.0%)	19915 (55.8%) - 17846 (56.2%)	30830 (92.6%) - 33718 (92.3%)	13485 (89.9%) - 15005 (89.2%)	529131 (57.8%) - 436691 (58.7%)	
Male	182829 (47.5%) - 120769 (46.7%)	46515 (26.3%) - 41434 (26.7%)	40795 (56.5%) - 41747 (57.0%)	17554 (29.2%) - 13458 (29.1%)	50706 (93.3%) - 42561 (93.6%)	18845 (44.4%) - 18519 (45.0%)	10501 (25.2%) - 10123 (26.0%)	15777 (44.2%) - 13898 (43.8%)	2453 (7.4%) - 2804 (7.7%)	1513 (10.1%) - 1823 (10.8%)	387488 (42.2%) - 307136 (41.3%)	
**Multi-Morbid people**	323153 (84.0%) - 238466 (92.2%)	176734 (99.8%) - 154922 (99.9%)	71562 (99.1%) - 73039 (99.7%)	60192 (100.0%) - 46243 (100.0%)	54318 (100.0%) - 45463 (100.0%)	42126 (99.3%) - 41020 (99.8%)	41413 (99.4%) - 38885 (99.8%)	35306 (98.9%) - 31628 (99.6%)	33283 (100.0%) - 36522 (100.0%)	14998 (100.0%) - 16828 (100.0%)	853085 (93.1%) - 723016 (97.2%)	
**Polymedicated people**	122956 (32.0%) - 84206 (32.5%)	110504 (62.4%) - 99185 (64.0%)	43115 (59.7%) - 44500 (60.8%)	51759 (86.0%) - 40528 (87.6%)	43829 (80.7%) - 38159 (83.9%)	30644 (72.2%) - 29788 (72.4%)	29416 (70.6%) - 28254 (72.5%)	20336 (57.0%) - 18717 (59.0%)	22739 (68.3%) - 25338 (69.4%)	12204 (81.4%) - 13710 (81.5%)	487502 (53.2%) - 422385 (56.8%)	
**Age (Mean)**	74.8 - 73.3	74.2 - 73.5	75.9 - 74.5	80.6 - 78.3	76.7 - 75.0	78.7 - 76.3	75.3 - 74.0	74.8 - 73.6	72.8 - 72.5	76.9 - 75.8	75.5 - 74.1	
**Age (n,%)**												916619 - 743827
[65,70)	121226 (31.5%) -	55687 (31.4%) -	17825 (24.7%) -	4711 (7.8%) - 5461 (11.8%)	10906 (20.1%) -	5578 (13.1%) - 7904	10981 (26.4%) - 12061	10135 (28.4%) -	12210 (36.7%) -	2919 (19.5%) -	252178 (27.5%) - 235001 (31.6%)	

	96106 (37.1%)	52749 (34.0%)	21292 (29.1%)		11395 (25.1%)	(19.2%)	(31.0%)	10472 (33.0%)	13773 (37.7%)	3788 (22.5%)		
[70,80)	162009 (42.1%) - 113532 (43.9%)	81567 (46.1%) - 72827 (47.0%)	31466 (43.6%) - 34163 (46.6%)	20490 (34.0%) - 20320 (43.9%)	24074 (44.3%) - 22198 (48.8%)	16968 (40.0%) - 19730 (48.0%)	19002 (45.6%) - 18753 (48.1%)	16359 (45.8%) - 15101 (47.6%)	16152 (48.5%) - 17933 (49.1%)	6499 (43.3%) - 7863 (46.7%)	394586 (43.1%) - 342420 (46%)	
[80,90)	86495 (22.5%) - 44749 (17.3%)	36744 (20.7%) - 27946 (18.0%)	20059 (27.8%) - 16495 (22.5%)	29143 (48.4%) - 18693 (40.4%)	17052 (31.4%) - 11113 (24.4%)	16937 (39.9%) - 12481 (30.4%)	10335 (24.8%) - 7616 (19.5%)	8316 (23.3%) - 5851 (18.4%)	4747 (14.3%) - 4682 (12.8%)	4916 (32.8%) - 4798 (28.5%)	234744 (25.6%) - 154424 (20.8%)	
[90,99]	15057 (3.9%) - 4313 (1.7%)	3089 (1.7%) - 1486 (1.0%)	2840 (3.9%) - 1287 (1.8%)	5848 (9.7%) - 1770 (3.8%)	2292 (4.2%) - 757 (1.7%)	2939 (6.9%) - 1005 (2.4%)	1326 (3.2%) - 531 (1.4%)	882 (2.5%) - 320 (1.0%)	174 (0.5%) - 134 (0.4%)	664 (4.4%) - 379 (2.3%)	35111 (3.8%) - 11982 (1.6%)	
**MEDEA:**												851564 - 720879
R	82025 (22.7%) - 54244 (21.6%)	33553 (20.0%) - 29758 (19.8%)	15314 (22.7%) - 15667 (22.0%)	12276 (23.5%) - 9279 (20.7%)	9282 (19.1%) - 7490 (17.1%)	8888 (23.7%) - 8089 (20.4%)	7512 (19.4%) - 7006 (18.6%)	6396 (19.8%) - 5650 (18.4%)	4888 (15.4%) - 5456 (15.4%)	2115 (15.3%) - 2350 (14.5%)	182249 (21.4%) - 144989 (20.1%)	
U1	67060 (18.6%) - 47089 (18.8%)	26625 (15.9%) - 23782 (15.8%)	10987 (16.3%) - 11839 (16.6%)	8247 (15.8%) - 7255 (16.2%)	7144 (14.7%) - 6673 (15.2%)	6691 (17.8%) - 7247 (18.3%)	5657 (14.6%) - 5535 (14.7%)	5534 (17.1%) - 5400 (17.6%)	4306 (13.5%) - 4983 (14.1%)	2540 (18.4%) - 2975 (18.3%)	144791 (17.0%) - 122778 (17.0%)	
U2	58542 (16.2%) - 41383 (16.5%)	27027 (16.1%) - 24586 (16.3%)	10976 (16.3%) - 11809 (16.6%)	8048 (15.4%) - 7160 (16.0%)	7442 (15.3%) - 7043 (16.0%)	5862 (15.6%) - 6555 (16.6%)	6062 (15.6%) - 5999 (15.9%)	5202 (16.1%) - 5056 (16.4%)	4964 (15.6%) - 5641 (15.9%)	2306 (16.7%) - 2806 (17.3%)	136431 (16.0%) - 118038 (16.4%)	
U3	57967 (16.0%) - 40622 (16.2%)	27628 (16.5%) - 25111 (16.7%)	10665 (15.8%) - 11607 (16.3%)	8076 (15.5%) - 7179 (16.0%)	8077 (16.6%) - 7486 (17.1%)	6116 (16.3%) - 6568 (16.6%)	6409 (16.5%) - 6301 (16.7%)	5192 (16.1%) - 5090 (16.6%)	5745 (18.1%) - 6362 (18.0%)	2347 (17.0%) - 2796 (17.2%)	138222 (16.2%) - 119122 (16.5%)	
U4	52828 (14.6%) - 37452 (14.9%)	27931 (16.6%) - 25006 (16.6%)	10526 (15.6%) - 10980 (15.4%)	7832 (15.0%) - 6990 (15.6%)	8361 (17.2%) - 7777 (17.7%)	5329 (14.2%) - 5952 (15.0%)	6730 (17.4%) - 6644 (17.6%)	5194 (16.1%) - 4974 (16.2%)	6003 (18.9%) - 6580 (18.6%)	2254 (16.3%) - 2724 (16.8%)	132988 (15.6%) - 115079 (16.0%)	
U5	42956 (11.9%) - 30178 (12.0%)	25050 (14.9%) - 22140 (14.7%)	8855 (13.2%) - 9253 (13.0%)	7775 (14.9%) - 6880 (15.4%)	8236 (17.0%) - 7432 (16.9%)	4686 (12.5%) - 5164 (13.0%)	6378 (16.5%) - 6282 (16.6%)	4805 (14.9%) - 4572 (14.9%)	5894 (18.5%) - 6379 (18.0%)	2248 (16.3%) - 2593 (16.0%)	116883 (13.7%) - 100873 (14.0%)	
**N. Chronic diseases, median [IQR]**	4.0 [2.0;5.0] - 5.0 [3.0;6.0]	6.0 [5.0;8.0] - 8.0 [6.0;9.0]	7.0 [5.0;8.0] - 8.0 [6.0;10.0]	9.0 [7.0;11.0] - 10.0 [8.0;12.0]	8.0 [7.0;10.0] - 10.0 [8.0;12.0]	7.0 [5.0;9.0] - 9.0 [6.0;11.0]	7.0 [5.0;9.0] - 9.0 [7.0;11.0]	7.0 [5.0;9.0] - 8.0 [6.0;11.0]	9.0 [7.0;10.0] - 10.0 [9.0;12.0]	11.0 [10.0;13.0] - 13.0 [11.0;15.0]	6.0 [4.0;8.0] - 7.0 [5.0;10.0]	

**N. Chronic diseases (n,%)**												916619 - 743827
0	25380 (6.6%) - 6584 (2.5%)	0 (0.0%) - 0 (0.0%)	0 (0.0%) - 0 (0.0%)	0 (0.0%) - 0 (0.0%)	0 (0.0%) - 0 (0.0%)	0 (0.0%) - 0 (0.0%)	0 (0.0%) - 0 (0.0%)	0 (0.0%) - 0 (0.0%)	0 (0.0%) - 0 (0.0%)	0 (0.0%) - 0 (0.0%)	25380 (2.8%) - 6584 (0.9%)	
1	36254 (9.4%) - 13650 (5.3%)	353 (0.2%) - 86 (0.1%)	628 (0.9%) - 198 (0.3%)	0 (0.0%) - 1 (0.0%)	6 (0.0%) - 0 (0.0%)	296 (0.7%) - 100 (0.2%)	231 (0.6%) - 76 (0.2%)	386 (1.1%) - 116 (0.4%)	0 (0.0%) - 0 (0.0%)	0 (0.0%) - 0 (0.0%)	38154 (4.2%) - 14227 (1.9%)	
[2, 5)	201282 (52.3%) - 108605 (42.0%)	30742 (17.4%) - 11516 (7.4%)	13785 (19.1%) - 7640 (10.4%)	1809 (3.0%) - 549 (1.2%)	1974 (3.6%) - 506 (1.1%)	6032 (14.2%) - 3276 (8.0%)	5751 (13.8%) - 2428 (6.2%)	6751 (18.9%) - 3105 (9.8%)	698 (2.1%) - 173 (0.5%)	12 (0.1%) - 1 (0.0%)	268836 (29.3%) - 137799 (18.5%)	
[5,10)	120673 (31.4%) - 126309 (48.8%)	132238 (74.7%) - 111530 (72.0%)	47338 (65.6%) - 44773 (61.1%)	32584 (54.1%) - 17095 (37.0%)	34571 (63.6%) - 19151 (42.1%)	25615 (60.4%) - 21911 (53.3%)	25450 (61.1%) - 20204 (51.9%)	21261 (59.6%) - 17305 (54.5%)	20365 (61.2%) - 13811 (37.8%)	3614 (24.1%) - 1583 (9.4%)	463709 (50.6%) - 393672 (52.9%)	
≥10	1198 (0.3%) - 3552 (1.4%)	13754 (7.8%) - 31876 (20.6%)	10439 (14.5%) - 20626 (28.2%)	25799 (42.9%) - 28599 (61.8%)	17773 (32.7%) - 25806 (56.8%)	10479 (24.7%) - 15833 (38.5%)	10212 (24.5%) - 16253 (41.7%)	7294 (20.4%) - 11218 (35.3%)	12220 (36.7%) - 22538 (61.7%)	11372 (75.8%) - 15244 (90.6%)	120540 (13.2%) - 191545 (25.8%)	
**N. of drugs, median [IQR]**	3.0 [1.0;5.0] - 3.0 [1.0;5.0]	6.0 [3.0;8.0] - 6.0 [4.0;8.0]	5.0 [3.0;8.0] - 5.0 [3.0;8.0]	8.0 [6.0;11.0] - 8.0 [6.0;11.0]	8.0 [5.0;10.0] - 6.0 [8.0;10.0]	7.0 [4.0;10.0] - 7.0 [4.0;9.0]	7.0 [4.0;10.0] - 7.0 [4.0;10.0]	5.0 [3.0;8.0] - 5.0 [3.0;8.0]	6.0 [4.0;9.0] - 6.0 [4.0;9.0]	8.0 [5.0;11.0] - 8.0 [5.0;10.0]	5.0 [2.0;8.0] - 5.0 [3.0;8.0]	
**N. of drugs (n,%)**												916619 - 743827
0	82698 (21.5%) - 41989 (16.2%)	9688 (5.5%) - 5355 (3.5%)	5408 (7.5%) - 3759 (5.1%)	2540 (4.2%) - 761 (1.6%)	2186 (4.0%) - 800 (1.8%)	2815 (6.6%) - 1463 (3.6%)	2366 (5.7%) - 1293 (3.3%)	3597 (10.1%) - 2199 (6.9%)	1473 (4.4%) - 1068 (2.9%)	597 (4.0%) - 265 (1.6%)	113368 (12.4%) - 58952 (7.9%)	
1	39683 (10.3%) - 28971 (11.2%)	6639 (3.7%) - 5728 (3.7%)	3467 (4.8%) - 3626 (5.0%)	505 (0.8%) - 353 (0.8%)	929 (1.7%) - 624 (1.4%)	1161 (2.7%) - 1257 (3.1%)	1246 (3.0%) - 1222 (3.1%)	2083 (5.8%) - 1822 (5.7%)	1159 (3.5%) - 1305 (3.6%)	210 (1.4%) - 283 (1.7%)	57082 (6.2%) - 45191 (6.1%)	
[2, 5)	139450 (36.2%) - 103534 (40.0%)	50256 (28.4%) - 44740 (28.9%)	20200 (28.0%) - 21352 (29.2%)	5388 (9.0%) - 4602 (10.0%)	7380 (13.6%) - 5880 (12.9%)	7802 (18.4%) - 8612 (20.9%)	8616 (20.7%) - 8192 (21.0%)	9676 (27.1%) - 9006 (28.4%)	7912 (23.8%) - 8811 (24.1%)	1987 (13.2%) - 2570 (15.3%)	258667 (28.2%) - 217299 (29.2%)	
[5,10)	108886 (28.3%) - 76263 (29.5%)	88542 (50.0%) - 79758 (51.5%)	32682 (45.3%) - 34143 (46.6%)	28606 (47.5%) - 23462 (50.7%)	26833 (49.4%) - 23058 (50.7%)	19941 (47.0%) - 20083 (48.8%)	18623 (44.7%) - 18442 (47.3%)	14879 (41.7%) - 13799 (43.5%)	16599 (49.9%) - 18510 (50.7%)	7090 (47.3%) - 8278 (49.2%)	362681 (39.6%) - 315796 (42.5%)	

≥10	14070 (3.7%) - 7943 (3.1%)	21962 (12.4%) - 19427 (12.5%)	10433 (14.5%) - 10357 (14.1%)	23153 (38.5%) - 17066 (36.9%)	16996 (31.3%) - 15101 (33.2%)	10703 (25.2%) - 9705 (23.6%)	10793 (25.9%) - 9812 (25.2%)	5457 (15.3%) - 4918 (15.5%)	6140 (18.4%) - 6828 (18.7%)	5114 (34.1%) - 5432 (32.3%)	124821 (13.6%) - 106589 (14.3%)	
**N. of visits, median [IQR]**	7.0 [3.0;12.0] - 7.0 [3.0;12.0]	10.0 [6.0;16.0] - 10.0 [6.0;16.0]	10.0 [6.0;17.0] - 10.0 [6.0;18.0]	20.0 [11.0;32.0] - 22.0 [11.0;34.0]	14.0 [9.0;23.0] - 15.0 [9.0;25.0]	12.0 [7.0;20.0] - 12.0 [7.0;21.0]	12.0 [7.0;20.0] - 12.0 [7.0;20.0]	11.0 [6.0;19.0] - 11.0 [6.0;19.0]	12.0 [8.0;19.0] - 12.0 [8.0;19.0]	15.0 [9.0;23.0] - 15.0 [9.0;24.0]	9.0 [5.0;16.0] - 10.0 [5.0;17.0]	
**N. of visits (n,%)**												916619 - 743827
0	36975 (9.6%) - 17397 (6.7%)	3722 (2.1%) - 2380 (1.5%)	1839 (2.5%) - 1335 (1.8%)	863 (1.4%) - 390 (0.8%)	763 (1.4%) - 395 (0.9%)	1147 (2.7%) - 780 (1.9%)	779 (1.9%) - 544 (1.4%)	1205 (3.4%) - 749 (2.4%)	390 (1.2%) - 311 (0.9%)	262 (1.7%) - 131 (0.8%)	47945 (5.2%) - 24412 (3.3%)	
1	23812 (6.2%) - 14629 (5.7%)	3649 (2.1%) - 3624 (2.3%)	1830 (2.5%) - 1922 (2.6%)	649 (1.1%) - 498 (1.1%)	629 (1.2%) - 568 (1.2%)	1051 (2.5%) - 1013 (2.5%)	749 (1.8%) - 672 (1.7%)	1043 (2.9%) - 866 (2.7%)	315 (0.9%) - 417 (1.1%)	157 (1.0%) - 187 (1.1%)	33884 (3.7%) - 24396 (3.3%)	
[2, 5)	77936 (20.3%) - 54464 (21.1%)	20693 (11.7%) - 18928 (12.2%)	9076 (12.6%) - 9669 (13.2%)	3064 (5.1%) - 2534 (5.5%)	3739 (6.9%) - 3050 (6.7%)	4606 (10.9%) - 4510 (11.0%)	3793 (9.1%) - 3558 (9.1%)	4310 (12.1%) - 3938 (12.4%)	2386 (7.2%) - 2866 (7.8%)	836 (5.6%) - 1104 (6.6%)	130439 (14.2%) - 104621 (14.1%)	
[5, 10)	117379 (30.5%) - 82678 (32.0%)	52731 (29.8%) - 46906 (30.3%)	20119 (27.9%) - 20721 (28.3%)	8287 (13.8%) - 5948 (12.9%)	10988 (20.2%) - 9136 (20.1%)	9698 (22.9%) - 9891 (24.1%)	10028 (24.1%) - 9391 (24.1%)	9029 (25.3%) - 8329 (26.2%)	8278 (24.9%) - 8991 (24.6%)	2812 (18.7%) - 3234 (19.2%)	249349 (27.2%) - 205225 (27.6%)	
≥10	128685 (33.4%) - 89532 (34.6%)	96292 (54.4%) - 83170 (53.7%)	39326 (54.5%) - 39590 (54.1%)	47329 (78.6%) - 36874 (79.7%)	38205 (70.3%) - 32314 (71.1%)	25920 (61.1%) - 24926 (60.6%)	26295 (63.1%) - 24796 (63.6%)	20105 (56.3%) - 17862 (56.3%)	21914 (65.8%) - 23937 (65.5%)	10931 (72.9%) - 12172 (72.3%)	455002 (49.7%) - 385173 (51.8%)	
